# Altered Profile of E1-S Transporters in Endometrial Cancer: Lower Protein Levels of ABCG2 and OSTβ and Up-Regulation of *SLCO1B3* Expression

**DOI:** 10.3390/ijms22083819

**Published:** 2021-04-07

**Authors:** Renata Pavlič, Suzana Vidic, Maja Anko, Tamara Knific, Tomaž Büdefeld, Kristina Marton, Maša Sinreih, Stefan Poschner, Walter Jäger, Snježana Frković-Grazio, Tea Lanišnik Rižner

**Affiliations:** 1 Institute of Biochemistry, Faculty of Medicine, University of Ljubljana, 1000 Ljubljana, Slovenia; renata.pavlic@mf.uni-lj.si (R.P.); suzana.vidic@mf.uni-lj.si (S.V.); maja.anko@mf.uni-lj.si (M.A.); tamara.knific@mf.uni-lj.si (T.K.); tomaz.budefeld@mf.uni-lj.si (T.B.); kristina.marton@mf.uni-lj.si (K.M.); masa.sinreih@mf.uni-lj.si (M.S.); 2 Department of Pharmaceutical Chemistry, University of Vienna, 1090 Vienna, Austria; stefan.poschner@univie.ac.at (S.P.); walter.jaeger@univie.ac.at (W.J.); 3 Department of Gynecological Pathology, Division of Gynaecology and Obstetrics, University Medical Centre Ljubljana, 1000 Ljubljana, Slovenia; snjezana.frkovicgrazio@kclj.si

**Keywords:** estrone-sulphate (E1-S), intracrinology, sulfatase pathway, E1-S transporters, ATP-binding cassette transporters, organic anion-transporting polypeptides, organic solute transporters

## Abstract

Endometrial cancer (EC) is associated with increased estrogen actions. Locally, estrogens can be formed from estrone-sulphate (E1-S) after cellular uptake by organic anion-transporting polypeptides (OATP) or organic anion transporters (OAT). Efflux of E1-S is enabled by ATP Binding Cassette transporters (ABC) and organic solute transporter (OST)αβ. Currently, 19 E1-S transporters are known but their roles in EC are not yet understood. Here, we analysed levels of E1-S transporters in Ishikawa (premenopausal EC), HEC-1-A (postmenopausal EC), HIEEC (control) cell lines, in EC tissue, examined metabolism of steroid precursor E1-S, studied effects of OATPs’ inhibition and gene-silencing on E1-S uptake, and assessed associations between transporters and histopathological data. Results revealed enhanced E1-S metabolism in HEC-1-A versus Ishikawa which could be explained by higher levels of OATPs in HEC-1-A versus Ishikawa, especially 6.3-fold up-regulation of OATP1B3 (*SLCO1B3*), as also confirmed by immunocytochemical staining and gene silencing studies, lower *ABCG2* expression and higher levels of sulfatase (STS). In EC versus adjacent control tissue the highest differences were seen for *ABCG2* and *SLC51B* (OSTβ) which were 3.0-fold and 2.1-fold down-regulated, respectively. Immunohistochemistry confirmed lower levels of these two transporters in EC versus adjacent control tissue. Further analysis of histopathological data indicated that *SLCO1B3* might be important for uptake of E1-S in tumours without lymphovascular invasion where it was 15.6-fold up-regulated as compared to adjacent control tissue. Our results clearly indicate the importance of E1-S transporters in EC pathophysiology and provide a base for further studies towards development of targeted treatment.

## 1. Introduction

Endometrial cancer (EC) is the most common gynaecological malignancy [1,2,3] with the majority of EC cases diagnosed in postmenopausal women. About 10% of EC cases are hereditary, but the rest are sporadic and can be traditionally classified into well-differentiated type 1 (70–80% of cases) and poorly differentiated type 2 (10–20% of cases), which is more aggressive and has poor prognosis [4]. EC type 1 is an estrogen-dependent disease where development and progression can be explained by exposure to estrogens of endogenous or exogenous origin, unopposed by progesterone or synthetic progestins, that increases the mitotic activity of endometrial cells and number of DNA replication errors leading to somatic mutations and a malignant phenotype [5,6]. EC type 2 has traditionally been considered estrogen independent [7,8], although no difference in estradiol levels has been found in tissue and plasma from patients with type 1 and type 2 EC which suggests that estrogens have roles also in EC type 2 [9,10]. More recent stratification of EC into four groups is based on the molecular characterizations [11,12].

In postmenopausal EC patients estrogens can be formed in peripheral sites (mainly adipose tissue) from inactive precursors including adrenal dehydroepiandrosterone (DHEA) and dehydroepiandrosterone-sulphate (DHEA-S) or ovarian androstenedione or from circulating estrone-sulphate (E1-S) [13,14,15]. In peripheral sites the most potent estrogen estradiol (E2) can be formed either by the aromatase pathway from DHEA-S and DHEA via androstenedione or testosterone by the action of sulfatase (STS), 3β-hydroxysteroid dehydrogenase (HSD3B), aromatase and reductive HSD17Bs or by the STS pathway from E1-S by the action of STS and reductive HSD17Bs. Our recent data and studies of other groups revealed that in EC the sulfatase pathway has a crucial role in formation of estrogens [15,16]. The sulfatase pathway depends on the circulating levels of E1-S which can be formed in adipose tissue from adrenal DHEA-S and DHEA [14]. As sulphated steroids cannot enter cells by simple diffusion uptake transporters are needed. Additionally, efflux transporters also have important roles, as they regulate intracellular concentration of E1-S.

Genes encoding E1-S uptake transporters belong to the solute carrier gene super family (SLC), families SLC21, SLC22 and SLC10 [17]. SLC21, better known as the solute carrier organic anion (SLCO) family, comprises genes encoding organic anion transporting polypeptides (OATP); eight of these are known to transport E1-S. The SLC22A subfamily comprises five organic anion transporters (OATs) which catalyse uptake of E1-S [18,19]. The SLC10 family comprises SLC10A6 which encodes solute anion transporter (SOAT) specific for transport of conjugated steroids and bile acids [20,21]. Genes encoding E1-S efflux transporters belong to the ATP binding cassette transporter family [17] where ABCC1, ABCC4, ABCC11 and ABCG2 mediate transport of E1-S [22]. These proteins are better known as multidrug resistance proteins (MRP) 1, 4, 8 and breast cancer resistance protein (BCRP) as they are up-regulated in chemoresistant types of cancer. SLC51 gene family includes two genes which encode organic solute transporters alfa and beta (OSTα and OSTβ). These proteins act as heterodimers [23,24] and mediate transport down the electrochemical gradient and can act as uptake or efflux transporters of E1-S [24]. Similarly to in breast cancer, where concentration of E1-S in cancer tissue surmounts E1-S concentrations in blood [25,26,27], EC tissue may also have higher E1-S levels, and thus OSTαβ would probably act as efflux transporter. Several *SLCO* genes show altered expression in breast cancer, while in endometrial cancer only increased expression of *SLCO1B3* was previously documented [13,28,29,30]. Although multispecific ABCs have been in the focus of anticancer research [22,31] and increased expression of *ABCG2* has been reported in breast cancer, data about their expression in endometrial cancer are limited to cell line Ishikawa only [32]. Uptake and efflux transporters have pivotal roles in local E2 formation via the STS pathway.

The aims of this study were manifold: (i) to examine the expression of all 20 genes encoding 19 E1-S transporters in model cell lines of EC and normal endometrium and in tissue samples of cancer and adjacent control endometrium, (ii) to examine protein levels of sulfatase in model cell lines, (iii) to evaluate the protein levels of the most up/down regulated genes in model cell lines of EC and paraffin tissue sections, (iv) to evaluate the ability of EC model cell lines to transport E1-S and to metabolize E1-S in the absence or presence of specific inhibitors or after silencing of specific genes for E1-S transporters, and (v) to assess the potential association of differentially expressed transporters with histopathological and clinical data.

## 2. Results

### 2.1. Fourteen Genes Encoding E1-S Transporters Are Differentially Expressed in Model Cell Lines Ishikawa and HEC-1-A

Based on the current published data, 19 transporter proteins have a capacity for E1-S transport (Table 1), including 14 uptake transporters, eight OATPs, five OATs, one SOAT, six efflux transporters, four ABC transporters, and heterodimeric organic solute transporter (OST) αβ.

**Figure 1:**
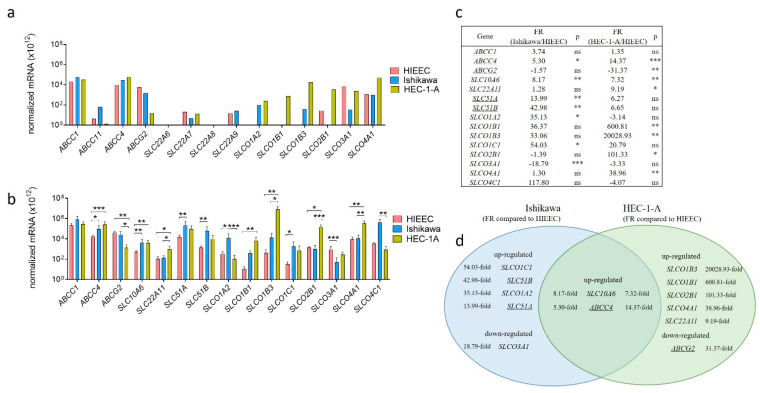
Expression of genes encoding E1-S transporters in model cell lines. (**a**) Expression of 14 genes involved in E1-S transport included in the PCR arrays for human drug transporters (PAHS-070Z) were examined in model cell lines, Ishikawa and HEC-1-A, and in control cell line, HIEEC. The data were normalized using normalization factor based on the expression of two most stably expressed genes (*ACTB* and *HPRT1*). (**b**) The expression of 15 ‘E1-S transporter genes’ was investigated in three independent experiments with two to three replicates, using newly designed primers. Statistical analysis was performed using Kruskal–Wallis test with Dunn’s multiple comparison test correction. (**c**) Based on normalized mRNA data, fold regulation (FR) was calculated for each gene in Ishikawa and HEC-1-A versus HIEEC. (**d**) Venn diagram representation of genes with significant difference in expression in Ishikawa and/or HEC-1-A compared to HIEEC. FR—fold regulation, ns—*p*-value > 0.05, * *p*-value ≤ 0.05, ** *p*-value ≤ 0.01, *** *p*-value ≤ 0.001, **** *p*-value ≤ 0.0001. Underlined genes are involved in E1-S efflux from cells. Data are shown as mean ± SD.

We first examined expression of genes encoding these E1-S transporters in the control cell line of proliferative endometrium, HIEEC and in model cell lines of EC, Ishikawa, a model of premenopausal and HEC-1-A, a model of postmenopausal EC (Figure 1). Preliminary expression analysis was performed by using Human drug transporter PCR array, which included assays for 14 E1-S transporters. Five genes (*ABCC11*, *SLC22A6*, *SLC22A7*, *SLC22A8*, *SLC22A9*) were not expressed or were very weakly expressed and there was no difference in their expression among cell lines (Figure 1a). Based on this preliminary expression data these five genes were excluded from further studies and additional six genes (*SLC10A6*, *SLC22A1*, *SLC51A*, *SLC51B*, *SLCO1C1*, *SLCO4C1*) were included, so we finally examined expression of 15 genes encoding E1-S transporters more in detail. Fourteen of these genes were differentially expressed in model cell lines (Figure 1b). Compared to HIEEC the expression of *ABCC4*, *SLC10A6*, *SLC51A*, *SLC51B*, *SLCO1A2* and *SLCO1C1* was more than two-fold up-regulated in Ishikawa and the expression of *ABCC4*, *SLC10A6*, *SLC22A11*, *SLCO1B1*, *SLCO1B3*, *SLCO2B1* and *SLCO4A1* was more than two-fold up-regulated in HEC-1-A. In Ishikawa and HEC-1-A, genes *SLCO3A1* and *ABCG2*, respectively, were more than two-fold down-regulated compared to HIEEC (Figure 1c). The highest difference in expression was seen for *SLCO1B3* and *ABCG2*—there were 20028.9-fold higher *SLCO1B3* and 31.4-fold lower *ABCG2* mRNA levels in HEC-1-A compared to HIEEC (Figure 1d).

### 2.2. Levels of OATP1B3 Are Higher in HEC-1-A Compared to Ishikawa

**Figure 2:**
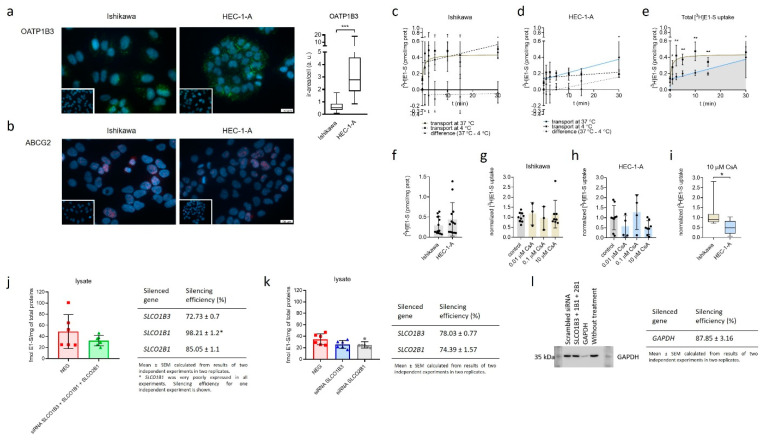
Immunocytochemical staining of OATP1B3 and ABCG2 and uptake of E1-S in Ishikawa and HEC-1-A. (**a**) Levels of OATP1B3 immunoreactivity in EC cell lines. Mann–Whitney U test after evaluation of 30 randomly chosen fields from three independent experiments (10 fields from each experiment). (**b**) Levels of ABCG2 immunoreactivity in EC cell lines. Representative pictures of ABCG2 staining are shown. Insets show corresponding cell lines incubated with normal rabbit (**a**) or mouse (**b**) serum. (**c**,**d**) Time-course of 16 nM [^3^H]E1-S uptake at 37 °C (total transport) and 4 °C (diffusion), measured in cell lysates in three individual experiments in duplicates. Transporter-mediated uptake of E1-S was calculated by subtraction of average values at individual temperatures. (**e**) Comparison of total E1-S uptake in Ishikawa and HEC-1-A over 30-min time-course. (**f**) Absolute uptake of E1-S after 30 min (Mann–Whitney U Test). (**g**,**h**) Normalized uptake of 16 nM [^3^H]E1-S after 30 min incubation with different concentrations of OATP inhibitor cyclosporine A (CsA). (**i**) E1-S uptake in the presence of 10 µM CsA. (Mann–Whitney U test). (**j**) Uptake of [^3^H]E1-S (2 min) after 48-h transient transfection with small-interfering RNAs targeting combination of genes *SLCO1B3*, *SLCO1B1* and *SLCO2B1* in HEC-1-A cell line (results of two independent experiments in three replicates (Mann–Whitney U test). (**k**) Effect of silencing of individual genes, *SLCO1B3* or *SLCO1B1* on 2 min [^3^H]E1-S uptake evaluated after 72-h siRNA transfection (two independent experiment in three replicates, Kruskal–Wallis with Dunn’s Multiple comparisons). (**l**) Western blot experiment confirming silencing of a control *GAPDH* (silencing efficiency (%) mean ± SEM, 87.85 ± 3.16) in two independent experiments in three replicates. In all experiments, silencing efficiency was calculated based on the expression of evaluated genes in NEG, cells treated with scrambled siRNA. Results of E1-S uptake were normalized to the total amount of proteins in cell lysates in individual treatments. Data are shown as mean ± SD. ir-area—immunoreactive area; a. u.—arbitrary unit, ns—*p*-value > 0.05, * *p*-value ≤ 0.05, ** *p*-value ≤ 0.01. *** *p*-value ≤ 0.001.

OATP1B3 *(SLCO1B3)* and ABCG2 with the highest difference in gene expression in Ishikawa and HEC-1-A compared to control cell line HIEEC were evaluated also at the protein levels using immunocytochemical staining (Figure 2). Protein OATP1B3 was detected in both EC cell lines with significantly higher levels seen in HEC-1-A (6.3-fold) compared to Ishikawa (Figure 2a). In HEC-1-A, strong signal for OATP1B3 was detected primarily in the cell membrane and only in some cells in the cytoplasm. In the Ishikawa cell line, OATP1B3 was primarily detected in the cytoplasm, with only a weak signal seen in the cell membrane.

In Ishikawa and HEC-1-A ABCG2 immunoreactivity was localized primarily to the nuclear area, whereas immunoreactive signal in cell membrane was below the level of detection (Figure 2b). Since the ABCG2 immunoreactivity in both cell lines was low and confined to the nuclear area, quantification of ABCG2 immunoreactivity was not performed.

### 2.3. Model Cell Line HEC-1-A Efficiently Metabolizes E1-S

**Figure 3:**
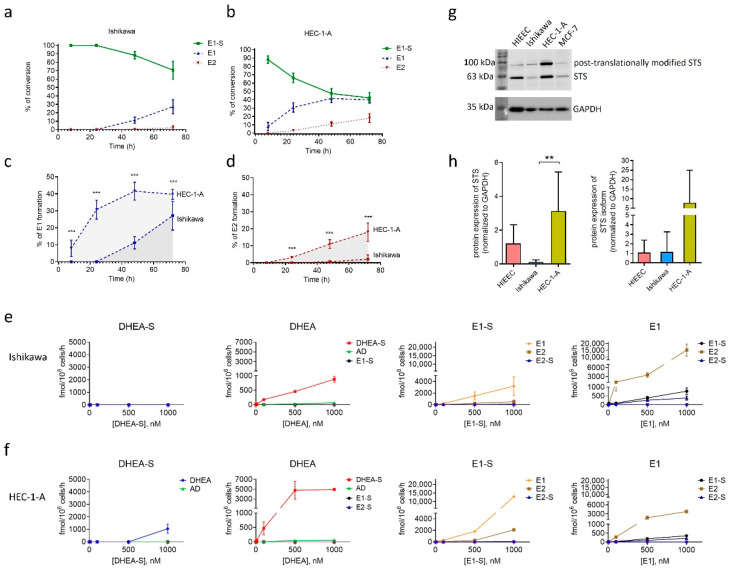
Metabolism of E1-S by Ishikawa and HEC-1-A cell lines evaluated using HPLC and LC-HRMS. Formation of metabolites E1 and E2 by Ishikawa (**a**) and HEC-1-A cells (**b**) after incubation with 16 nM [^3^H]E1-S was evaluated by HPLC. The levels of individual metabolites formed after 8 h, 24 h, 48 h and 72 h are shown as percentages (%) of total products. Comparison of E1-S metabolism to E1 (**c**) and E2 (**d**) in HEC-1-A compared to Ishikawa. Three independent experiments (Mann–Whitney U test). (**e**,**f**) Formation of metabolites (AD, DHEA, DHEA-S, E1, E1-S, E2 and E2-S) was evaluated by LC-HRMS in medium of Ishikawa and HEC-1-A cells 48 h after addition of 10, 100, 500 or 1000 nM DHEA-S, DHEA, E1-S or E1 in two replicates for individual treatment. (**g**) STS protein levels in HIEEC, Ishikawa, HEC-1-A and a positive control MCF-7. Cropped sections of one representative membrane are shown. The full-length coomassie-stained gels, Ponceau S-stained membranes, full-length membranes after chemiluminescent detection of STS and GAPDH, and densitometric readings are presented in Supplementary File, Figure S1. (**h**) STS levels in cell lines HIEEC, Ishikawa and HEC-1-A. GAPDH was used as a loading control. The levels of STS were determined in four independently isolated protein samples from four cell lines. Western blot was repeated independently two times. The levels of STS in each sample were normalized to its loading control. One-way ANOVA with Bonferroni’s Multiple Comparison test was used to determine statistical significance. Data are shown as mean ± SD. ** *p*-value ≤ 0.01, *** *p*-value ≤ 0.001.

Next, E1-S metabolism studies were performed in cell lines Ishikawa and HEC-1A to evaluate functionality of E1-S transporters and STS enzyme (Figure 3). The cells were treated with [^3^H]E1-S (16 nM) and the formation of metabolites (E1 and E2) was monitored over a time course of 72 h. As expected [33], we observed high conversion of E1-S in HEC-1-A associated with increased levels of E1, and also E2, in the medium (Figure 3b). The levels of E1 were measurable eight hours after the treatment and reached the steady state after 48 h. In contrast, the formation of E2 steadily increased over the 72 h time course. In Ishikawa cells, a lower percentage of E1-S was metabolized to E1. The levels of E1 were measurable 48 h after treatment and increased steadily within the 72 h. The formation of E2 was hardly detectable only after 72 h (Figure 3a). The formation of E1 and E2 was significantly higher in HEC-1-A compared to Ishikawa (Figure 3c,d).

To confirm the importance of the STS pathway and the difference in steroid metabolism we next also examined metabolism of DHEA-S, DHEA, E1-S and E1 in both EC cell lines by liquid chromatography–high-resolution mass spectrometry (LC-HRMS) analysis (Figure 3e,f). Ishikawa and HEC-1-A cells were treated with 10–1000 nM DHEA-S, DHEA, E1-S or E1 for 48 h and the presence of ten metabolites (androstenedione (AD), DHEA, DHEA-S, E1, E1-S, E2, estradiol sulphate (E2-S), estradiol glucuronide (E2-G), estriol (E3), testosterone (T)) were analysed in the cell medium. LC-HRMS results confirmed higher levels of E1 and E2 formation in HEC-1-A as compared to Ishikawa when the cells were treated with E1-S. When the cells were treated with E1, there was a higher formation of E2 and E1-S in Ishikawa compared to HEC-1-A cells. These results indicate that both EC cell lines markedly differ in the presence and/or actions of E1-S uptake and efflux transporters and also enzymes STS and reductive HSD17B, which are responsible for the formation of E2, and sulfotransferase SULT1E1, which catalyses conjugation of estrogens. Based on the LC-HRMS results, higher levels of OATP transporters and lower levels of ABC transporters are expected in HEC-1-A compared to Ishikawa.

Our results confirmed that in model EC cell lines precursors of the aromatase pathway, DHEA-S and DHEA, were not transformed to estrogens. DHEA-S was metabolized only in HEC-1-A cells to a small extent to DHEA and AD only at the highest, non-physiological concentration. DHEA was predominately metabolized to DHEA-S in both EC cell lines with higher levels seen in HEC-1-A. As E1 and E2 were not formed in EC cell lines when these were treated with DHEA-S or DHEA the results confirm our previous observations [15] that in EC the sulfatase pathway has the pivotal role in E2 formation.

### 2.4. Model Cell Lines HIEEC, Ishikawa and HEC-1-A Differ in Protein Levels of STS

After E1-S enters the cells, metabolism proceeds via the sulfatase pathway. In our previous study [33] we have already shown that gene *STS* is expressed in both EC model cell lines, Ishikawa and HEC-1-A, as well as in the control cell line of proliferative endometrium, HIEEC. Here, we confirmed STS activity (Figure 3a–f) and the expression of STS also at the protein level (Figure 3g,h) using specific and validated anti-STS antibodies [15]. By Western blot analysis, we observed two signals for STS, the first at around 63 kDa corresponds to STS, and the second signal at higher masses (around 100 kDa) probably corresponds to the N-glycosylated isoform. STS contains four potential N-glycosylation motifs and two of these sites (N47 and N259) are most probably used for N-linked glycosylation [34]. Our data show that STS levels differ between individual cell lines; the highest protein levels of STS and its glycosylated isoform were observed in HEC-1-A and were significantly higher (*p* = 0.0022) as compared to Ishikawa. These results support our previous observations of enhanced E1-S metabolism and increased E1 and E2 formation in HEC-1-A compared to Ishikawa [33], which were also confirmed in this study (Figure 3a–f).

### 2.5. Model Cell Lines Ishikawa and HEC-1-A Differ in E1-S Uptake

The bottleneck in E1-S metabolism is the cellular uptake of E1-S. In our transport studies Ishikawa and HEC-1-A were exposed to 16 nM [^3^H]E1-S for over 30-min time course (Figure 2c–f). In Ishikawa, total E1-S uptake steeply increased for 2.5 min, then slowed down and reached a plateau at around 10 min. In contrast, total E1-S uptake steadily increased for 30 min in HEC-1-A and did not saturate. In Ishikawa cells, E1-S transport at 4 °C and at 37 °C showed very similar profiles and transporter-mediated E1-S uptake was insignificant. In HEC-1-A the transport at 4 °C changed very little in evaluated time course and the transporter-mediated E1-S uptake steadily increased for 30 min without reaching a plateau. Comparison of total E1-S uptake in both EC cell lines thus revealed important difference in the profiles, which may lead to higher E1-S uptake and metabolism observed in HEC-1-A cells.

The transport of E1-S in Ishikawa and HEC-1-A was also studied in the presence of cyclosporine A (CsA) which acts as a general inhibitor of OATP transporters [35] (Figure 2g–i). Comparison between both cell lines revealed significantly higher inhibition of E1-S uptake in HEC-1-A versus Ishikawa when the cells were treated with 10 µM CsA and no significant differences when both cell lines were treated with 0.01 or 0.1 µM CsA. Higher effects of CsA treatment seen in HEC-1-A indicates that in these cells OATPs have more important roles in E1-S uptake as compared to Ishikawa cells.

### 2.6. Silencing of SLCO1B3, SLCO1B1 and SLCO2B1 Affects the Uptake of E1-S in HEC-1-A

To further evaluate the importance of OATP transporters in E1-S uptake, we performed siRNA-mediated silencing of three most up-regulated genes in HEC-1-A, *SLCO1B3, SLCO1B1* and *SLCO2B1* (20028.9-fold, 600.8-fold, and 101.3-fold up-regulation in HEC-1-A versus HIEEC, respectively) and examined [^3^H]E1-S transport (Figure 2j,k). Results revealed 33.4% lower levels of E1-S in cell lysates of HEC-1-A cells after silencing these three E1-S uptake transporter genes compared to negative control (cells treated with scrambled siRNA). In the next experiments, genes *SLCO1B3* and *SLCO2B1* were silenced individually. Gene *SLCO1B1* was less expressed as compared to *SLCO1B3* and *SLCO2B1* and was thus not included. Silencing of *SLCO1B3* revealed 25.4% lower levels of E1-S and silencing of *SLCO2B1* 27.7% lower levels of E1-S in cell lysates compared to negative control, which all confirm decreased uptake of E1-S in these cells. Both genes, *SLCO1B3* and *SLCO2B1,* are thus crucial for E1-S uptake in HEC-1-A cells. Our experimental data thus support importance of OATP2B1 and OATP1B3 in E1-S uptake in HEC-1-A cell line.

### 2.7. Three Genes Encoding E1-S Transporters Are Differentially Expressed in EC Tissue

**Figure 4:**
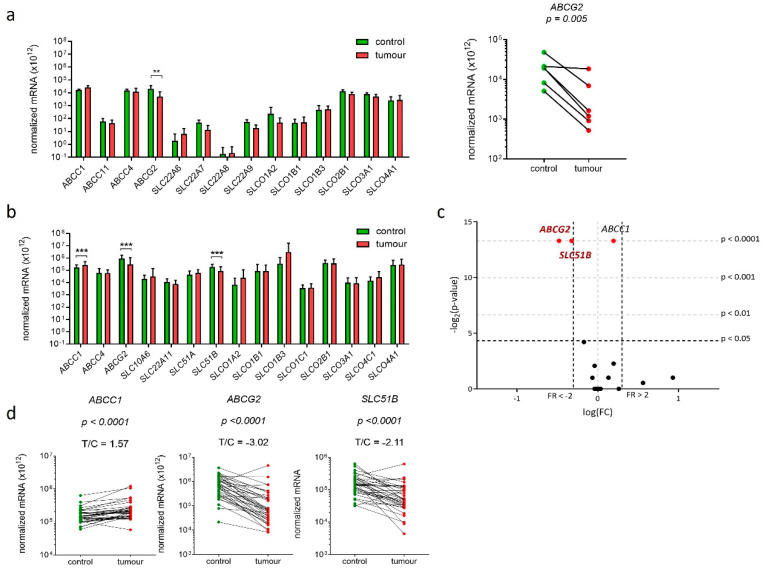
Expression of genes encoding E1-S transporters in 36 paired samples of EC and adjacent control tissue. (**a**) Normalized mRNA expression level of 14 genes encoding E1-S transporters (Human Drug transporters PCR Array PAHS-070Z) in 6 paired tissue samples (Wilcoxon matched pairs with Bonferroni’s multiple comparison). (**b**) Normalized mRNA expression analysis of 15 genes encoding E1-S transporters in 36 paired tissue samples (Wilcoxon matched pairs with Bonferroni’s multiple comparison tests) in EC compared to control tissue. (**c**) Volcano plot shows the most significantly down-regulated and the most significantly up-regulated genes. (**d**) Before-and-after graphs show the normalized mRNA levels for genes *ABCC1, ABCG2* and *SLC51B* in 36 paired tissue samples. Data are shown as a mean ± SD on the logarithmic scale. ** *p*-value ≤ 0.01, *** *p*-value ≤ 0.001.

Experimental data obtained in model cell lines indicated that E1-S transporters may have roles in pathophysiology of EC thus we next examined expression of all 20 genes encoding 19 E1-S transporters in EC and adjacent control endometrium (Figure 4). Preliminary expression analysis was performed on six pairs of EC and adjacent control endometrium using Human drug transporter PCR array, which included assays for 14 E1-S transporters. Six of these genes were not expressed or were very weakly expressed (*ABCC11*, *SLC22A6*, *SLC22A7*, *SLC22A8*, *SLC22A9, SLCO1B1*), and among the other eight genes, only gene *ABCG2* showed significant difference in expression in cancer versus adjacent control tissue (Figure 4a). Based on this preliminary data, genes that were not expressed or were expressed in low levels were excluded, with the exception of gene *SLCO1B1,* which was included in the further analysis, since it is closely related to *SLCO1B3* gene [36]. Six additional genes (*SLC10A6*, *SLC22A1*, *SLC51A*, *SLC51B*, *SLCO1C1*, *SLCO4C1*) were included to finally examine the expression of 15 genes encoding E1-S transporters in the total of 36 pairs of cancer and adjacent control tissue. Statistically significant changes were seen for three genes *ABCC1*, *ABCG2* and *SLC51B*; *ABCG2* and *SLC51B* were significantly (3.2-fold and 2.1-fold, respectively) down-regulated and *ABCC1* was significantly (1.6-fold) up-regulated in EC tissue compared to adjacent control tissue (Figure 4b–d). These genes are involved in E1-S efflux from cells and therefore their changed expression may influence intracellular E1-S levels.

### 2.8. Genes Encoding E1-S Transporters Are Differentially Expressed in EC Tissue Samples from Postmenopausal Patients

**Figure 5:**
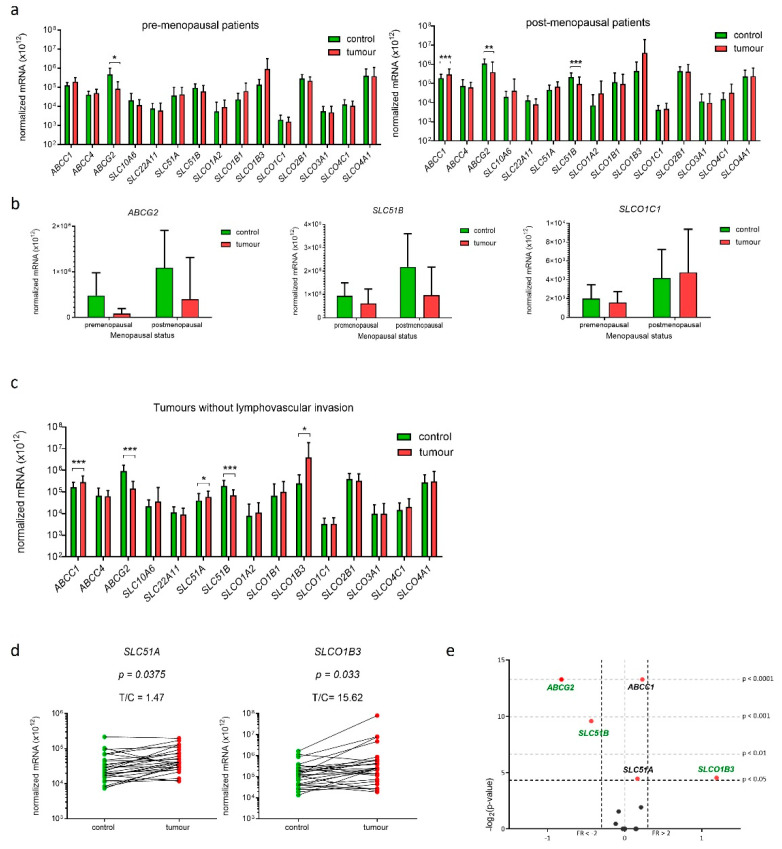
Expression of genes encoding E1-S transporters in paired tissue samples from premenopausal and postmenopausal patients and patients without lymphovascular invasion. (**a**) Expression in tissue samples from premenopausal patients (*n* = 11) and tissue samples from postmenopausal patients (*n* = 25). (Wilcoxon matched pairs with Bonferroni‘s multiple comparison test). (**b**) Effects of menopausal status on expression of three genes: *ABCG2*, *SLC51B* and *SLCO1C1* (two-way ANOVA). (**c**) Expression of genes in samples from patients without lymphovascular invasion (*n* = 27) (Wilcoxon matched pair tests with Bonferroni multiple comparison correction). (**d**) Before-and-after graphs show the normalized mRNA levels of *SLC51A* and *SLCO1B3* in 27 paired tissue samples. (**e**) Volcano plot shows fold change (FC) of normalized mRNA levels in tumours vs. controls. Data are shown as mean ± SD, * *p*-value ≤ 0.05, ** *p*-value ≤ 0.01, *** *p*-value ≤ 0.001.

We next stratified patients according to their menopausal status. In premenopausal samples (*n* = 11) (Figure 5a) statistically significant changes were observed for only one gene, *ABCG2*, which was 5.9-fold down-regulated in cancer compared to adjacent control tissue. In postmenopausal samples (*n* = 25) *ABCG2* and *SLC51B* were 2.8-fold and 2.2-fold down-regulated, respectively, and *ABCC1* was 1.6-fold up-regulated. Additional analysis using two-way ANOVA confirmed that menopausal status affected expression of *ABCG2*, *SLC51B* and also *SLCO1C1* (Figure 5b). Since the expression of *SLCO1C1* was affected only by the menopausal status (it was up-regulated in postmenopausal versus premenopausal patients) and not by the disease itself, this gene was excluded from further analyses. The differential expression of E1-S transporters in postmenopausal women supports importance of the local estrogen formation and intracrine estrogen action in women in the last decades of their lives.

### 2.9. Gene SLCO1B3 Is Up-Regulated in EC Tissue Samples from Patients without Lymphovascular Invasion

We then examined whether the expression of genes encoding E1-S transporters has any prognostic value. We stratified patients according to the clinical data (FIGO stage) and the histopathological data (i.e., tumour histological grade, depth of myometrial invasion and presence of lymphovascular invasion (LVI)). In patients with FIGO stage IA (*n* = 25), patients with low-grade EC (*n* = 25) and patients with ≤50% invasion into the myometrium (*n* = 26) in addition to genes *ABCC1*, *ABCG2*, and *SLC51B* also gene *SLC51A* was differentially expressed—about 1.6-fold up-regulated. The product of the *SLC51A* gene is a transmembrane protein OSTα which has to dimerize with OSTβ to form functional transporter. Interestingly, gene *SLC51A* was up-regulated, while gene *SLC51B* was about 2-fold down-regulated in EC versus control tissue from patient with FIGO stage IA, low-grade tumour and tumour with ≤50% invasion into myometrium, which may affect the levels of the active OST heterodimer.

Stratification according to the presence of LVI, which is considered important prognostic factor, revealed that E1-S transporters are differentially expressed only in endometrial tissue from patients without LVI (*n* = 27) (Figure 5c). In these patients, five genes were differentially expressed, in addition to *ABCG2*, *SLC51B* and *ABCC1* also genes *SLC51A* and *SLCO1B3*. Similarly to in patients with FIGO IA stage, low-grade tumours and patients with ≤50% invasion into the myometrium also in patients without LVI gene *SLC51A* was up-regulated (1.5-fold), while gene *SLC51B* was down-regulated (2.7-fold) in EC versus control tissue. Interestingly, gene *SLCO1B3,* which encodes uptake transporter OATP1B3 was 15.6-fold up-regulated only in cancer tissue of these patients without LVI and this may increase uptake of E1-S and thus also its intracellular concentration.

In EC tissue samples from patients with higher FIGO stages (IB-IV) (*n* = 11) only gene *SLC22A11* was 2.6-fold down-regulated. In patients with deep invasion into myometrium (>50%) (*n* = 9) genes encoding E1-S transporters were not differentially expressed, and in high-grade tumours (*n* = 10), we saw 2–3-fold down-regulation of four genes; *ABCG2*, *SLC10A6*, *SLC22A11* and *SLC51B.* Genes *SLC22A11* and *SLC10A6* encode uptake transporters SOAT and OAT4, respectively, where decline in expression of these uptake transporters in high-grade tumours may reduce intracellular concentrations of E1-S. The groups of patients with FIGO IB-V, high-grade cancers, patients with LVI and patients with deep >50% invasion into myometrium included in our study were small, thus data obtained in these groups have to be considered with caution and additional studies in larger groups of patients are needed. Finally, analysis using two-way ANOVA revealed that among all clinical and histopathological data, only grade significantly affected expression of *SLC51B* gene with lower levels seen in high-grade tumours (Supplementary Figure S2).

### 2.10. Protein Levels of Efflux Transporters ABCG2, OSTβ and OATP1B3 Are Significantly Lower in EC Tissue Compared to Adjacent Control Tissue

**Figure 6:**
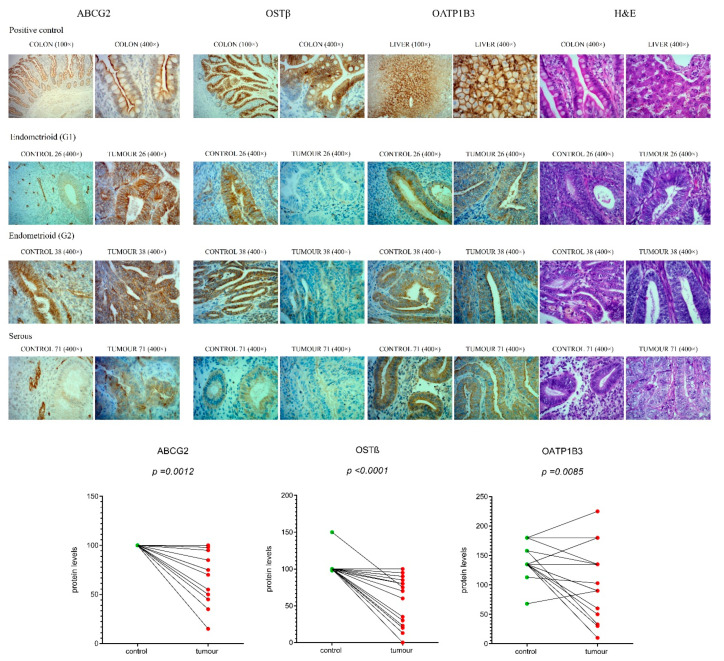
Immunohistochemical staining for ABCG2, OSTβ and OATP1B3 in EC and adjacent control endometrium. Representative control endometrium (C26, C38, C71), endometrioid grade 1 tumour (T26), endometrioid grade 2 tumour (T38), serous tumour (T71), colon and liver paraffin sections (positive controls), were stained with anti-ABCG2, anti-OSTβ and anti-OATP1B3 antibodies, and with haematoxylin and eosin (H&E) staining. Magnifications of 100× and 400× are shown. Protein levels of ABCG2, OSTβ and OATP1B3 in EC compared to adjacent control tissue (*n* = 20) are shown as before-and-after graphs (paired *t*-test). The same trends in protein levels were also seen for immunohistochemical analysis of all 52 samples, paired and unpaired (figure not shown).

For the most significantly differentially expressed genes in EC tissue samples, *ABCG2* and *SLC51B,* we also examined protein levels, using validated antibodies (Table 2, Figure S2, Table S1). We performed IHC on tissue micro arrays (TMA), which included paraffin section of 46 EC samples and 26 control tissue samples (Figure 6). Staining for ABCG2 showed membranous as well as cytoplasmic reaction both in the tumour samples and adjacent control endometrium. The same pattern with less intense staining was seen for control secretory endometrium and distinct strong reaction was observed in the endothelial cells. Staining for OSTβ indicated mainly cytoplasmic reaction that was present in all control endometrium samples and in the majority of the tumour samples. The staining in the tumour samples was heterogeneous, with some indication of distinct luminal accumulation of this protein. The control secretory endometrium showed less intense staining compared to control proliferative or inactive control endometrium. Consistently endothelial cells showed distinct membranous reaction and also inflammatory cells were positive. Results of the immunohistochemical scoring revealed significantly lower (*p* < 0.01) levels of OSTβ and ABCG2 in EC compared to the adjacent control tissue (Figure 6). The same trends were seen when all EC tissue samples were compared to control tissue.

Additionally, for the gene *SLCO1B3*, which has the highest up-regulation in tissue from patients without LVI, we examined the protein levels, using validated antibodies against OATP1B3 (Table 2, Figure S2, Table S1). Staining for OATP1B3 showed distinct cytoplasmic reaction in all samples of endometrium, intense membranous staining in liver as positive control and positive endothelial cells in all samples. In endometrium samples we also observed that in the cases with less intense staining there was distinct luminal accumulation. Although antibodies against OATP1B3 showed membranous staining in the liver, in EC and adjacent control tissue, immunoreactivity was observed mainly in cytoplasm, which calls into question the functionality of this transporter. Further immunohistochemical evaluation revealed less difference between EC and adjacent control tissue, as compared to ABCG2 and OSTβ, but still significantly decreased levels of OATP1B3 (*p* < 0.05) in EC (Figure 6), which is not in line with qPCR data.

These profiles of staining were seen in postmenopausal women with low-grade cancers, patients with FIGO stage IA, without LVI and with ≤50% invasion into the myometrium (Table 3). Further statistical analysis using two-way ANOVA showed no effects of the menopausal status on the protein levels of ABCG2, OSTβ and OATP1B3. In two-way ANOVA, the effects of menopausal status on *ABCG2* and *SLC51B* (OSTβ) expression were thus seen only at the mRNA levels (Figure 5b), which can be explained by the fact that immunohistochemistry is a semiquantitative method and could at our sample size (12 premenopausal and 40 postmenopausal tissue samples) primarily reveal only very large differences between groups.

## 3. Discussion

EC is an estrogen-dependent disease with the majority of cases diagnosed in postmenopausal women [40]. In women after menopause, the ovaries cease to act; thus, in that period of life, estrogens are formed only in peripheral sites from inactive adrenal precursors DHEA-S, DHEA and from circulating levels of E1-S. The precursor steroid E1-S can be formed mainly in adipose tissue but also in several other peripheral tissues [41]. In EC patients, increased serum concentrations of E1-S have been reported [42]. Although E1-S cannot enter cells by simple diffusion the importance of E1-S uptake and efflux for local estrogen formation and action has not yet been studied in EC [13]. We thus evaluated expression and function of E1-S transporters in model cell lines of EC and tissue samples of EC and adjacent control endometrium.

Our data show that 14 genes encoding E1-S transporters were differentially expressed in model cell lines of EC, Ishikawa and HEC-1-A (Figure 1), which suggests that these transporters have important roles in EC pathophysiology. We have shown previously [33], and confirmed here (Figure 3), that cell line HEC-1-A, a model of moderately/well differentiated EC from postmenopausal patient, has a higher capacity for E1-S metabolism, which depends on E1-S uptake transporters and STS, as compared to cell line Ishikawa—a model of well-differentiated EC from a premenopausal patient. The results of our current study further revealed that both EC cell lines differ not only in E1-S metabolism but also in E1 metabolism to E1-S and E2, with higher levels of these two metabolites seen in Ishikawa (Figure 3). Most importantly, we confirmed here again [15] that in EC sulfatase pathway is pivotal for E2 formation, while DHEA-S and DHEA cannot serve as precursors for E2, and the balance between hydrolysis of DHEA-S and sulfation of DHEA is clearly shifted to sulfation (Figure 3e,f).

The observed increased metabolism of E1-S in HEC-1-A cells versus control cell line HIEEC may be explained by differences in E1-S uptake due to up-regulation of E1-S transporter *SLCO1B3* (20028-fold) (Figure 1) and correspondingly its higher protein levels (6.3-fold) (Figure 2). OATP transporters are transmembrane proteins, and thus membrane localization of OATP1B3 (Figure 2) supports the functionality of this transporter in HEC-1-A cells. Functionality of OATP1B3 in these cells was additionally confirmed by gene silencing studies followed by E1-S transport studies (Figure 2j,k). Additionally, the down-regulation (31-fold) of efflux transporter *ABCG2* (Figure 1) and low protein levels of ABCG2 (Figure 2) support enhanced E1-S uptake in HEC-1-A cells.

Additionally, the lack of ABCG2 in cell membranes with immunoreactivity primarily seen in cell nucleus indicates that this transporter may not be functional or may be involved in some other pathological processes. Nuclear localization of ABCG2 has previously been reported in glioblastoma multiforme cells [43] and also in lung cancer cells [44], where decreased ABCG2 expression was connected with down-regulation of E-cadherin and enhanced motility of cancer cells.

Increased metabolism of E1-S in HEC-1-A compared to the Ishikawa cell line is also supported by E1-S transport (Figure 2c–f). Different E1-S uptake profiles were observed in HEC-1-A versus Ishikawa. E1-S uptake in Ishikawa reached a plateau soon after E1-S addition, while in HEC-1-A it continuously increased during the time investigated, potentially leading to increased intracellular levels and enhanced E1-S metabolism seen in HEC-1-A versus Ishikawa.

The importance of E1-S uptake was confirmed when these cells were treated with CsA, a general inhibitor of OATP transporters. Inhibition of E1-S uptake was more pronounced in HEC-1-A compared to Ishikawa (Figure 2g–i) which indicates higher levels of OATP transporters in HEC-1-A. Indeed, genes *SLCO1B3*, *SLCO1B1* and *SLCO2B1* encoding OATP transporters were highly up-regulated in HEC-1-A versus HIEEC (Figure 1). Simultaneous silencing of these three genes in HEC-1-A significantly affected the uptake of E1-S (Figure 2j) with lower levels of E1-S seen in cell lysate. Further silencing of individual genes, *SLCO1B3* and *SLCO2B*, further confirmed the importance of these OATP transporters in E1-S transport in HEC-1-A cell line.

Differences in OATP1B3 and ABCG2 expression at the mRNA and protein levels, differences in E1-S transport and CsA inhibition profiles, as well as SLCO1B1/1B3/2B1 silencing may all explain increased capacity for E1-S metabolism in HEC-1-A cells as compared to Ishikawa. Additionally, increased E1-S metabolism to E1 and E2 [33] (Figure 3a–f), which is catalyzed by STS and reductive HSD17Bs, is also supported by significantly increased protein levels and activity of STS in HEC-1-A versus Ishikawa (Figure 3g,h). On the other hand, increased E1 metabolism to E1-S and E2 in Ishikawa compared to HEC-1-A could be explained by insignificantly higher expression of *SULT1E1* and lower expression of *HSD17B2* in Ishikawa versus HEC-1-A and thus changed balance between reductive HSD17Bs and oxidative HSD17B2, as reported in our previous study [33]. Overall, our data in the selected model cell lines support importance of intracrine estrogen formation in postmenopausal women.

In tissue samples, too, the experimental data show that genes encoding E1-S transporters are differentially expressed mainly in postmenopausal patients. In these patients estrogens can only be formed in peripheral tissues from precursor steroids DHEA-S, DHEA and E1-S [13,15], and thus intracrine action of estrogens has a pivotal role. In EC tissue samples, only genes encoding efflux transporters were differentially expressed; *ABCG2* and *SLC51B* were more than 2-fold down-regulated, and *ABCC1* was less than 2-fold up-regulated (Figure 4). Additionally, IHC staining in 46 EC samples and 26 control samples revealed significantly lower protein levels of ABCG2 and OSTβ in cancer tissue (Figure 6). As expected, staining for ABCG2 was membranous and to lower extent cytoplasmic, while staining for OSTβ was seen mainly in cytoplasm indicating lack of functionality of this protein. More than two-fold decreased expression of genes *ABCG2* and *SLC51B* and lower protein levels of ABCG2 and OSTβ together with cytoplasmic localization probably surmounts a minor increase in *ABCC1* expression. Lower levels of two efflux transporters may thus decrease excretion of E1-S and may consequently sustain high intracellular E1-S concentration, which may indirectly facilitate activation to E2.

Expression analysis implies that E1-S transporters have roles only in postmenopausal EC, with good prognosis, as shown for patients with FIGO IA stage, low-grade tumours and patients with ≤50% invasion into the myometrium. In these patients with good prognosis, we observed difference in expression of four genes. Interestingly, genes *SLC51B* and *SLC51A* showed opposite regulation in EC samples from these patients, *SLC51B* was down-regulated and *SLC51A* was up-regulated. *SLC51B* encodes OSTβ, a single transmembrane domain polypeptide, while *SLC51A* encodes the 7-transmembrane domain, where protein-heterodimerization is required for delivery of the functional transporters to the plasma membrane [24]. Altered regulation of *SLC51B* and *SLC51A* would thus affect the concentrations of the active transporter and indirectly different physiological/pathophysiological pathways. Studies in knock out mice revealed that heterodimer OSTαβ has roles in bile acids, steroid (E1-S, DHEA-S and pregnane sulphate) and prostaglandin E2 homeostasis [24], where localization to steroid-rich tissues, including uterus, indicate that OSTαβ also contribute to transport of steroidal compounds [45,46]. Genes *SLC51A* and *SLC51B* are usually coregulated, and we found no report on inverse regulation of these genes.

There are very limited data available about expression of *SLC51B* and *SLC51A* in cancer [45]. Interestingly, OATP1B3 and OATP4C1 are the only E1-S transporters that have previously been investigated in EC tissue [28,47]. The levels of OATP4C1 were found to be higher in EC compared to normal endometrial tissue where the expression of *SLCO4C1* correlates positively with disease progression [47]. We observed no difference in *SLCO4C1* expression in EC versus adjacent control tissues and found no association with any of the prognostic factors probably due to the fact that our cohort included mostly low-grade EC samples (26 low grade and 10 high grade). OATP1B3 was previously associated with increased disease-free survival of patients with stage III endometrial cancer, which was explained by increased uptake of paclitaxel and thus better outcome of treatment [28]. OATP1B3 is not specific only for E1-S but transports a variety of endogenous and exogenous organic anions, including taxanes. Our study revealed contradictory data, while IHC analysis suggested that a large part of the protein is not functional as it is not localized to the membranes, qPCR data showed significant (15.6-fold) up-regulation of *SLCO1B3* in patients without LVI and thus supported association with increased disease-free survival. Additionally, the analysis of TCGA data (TCGA cohort of 1227 patients with endometrioid EC, at The cBioPortal for Cancer Genomics; www.cbioportal.org, accessed on 16 October 2020) supported our qPCR data and showed that *SLCO1B3* mRNA levels are higher (z-score median 0.57) in EC versus control tissue and correlate with longer disease free survival of patients.

In contrast to model cell lines, where 14 genes were differentially expressed, in tissue samples there were only two genes down-regulated and one gene up-regulated. When we compare model cell lines with tissue samples, the highest similarity can be seen between HEC-1-A cell line (a model of postmenopausal EC) versus control cell line HIEEC and EC tissue from patients without LVI versus adjacent control tissue. In both cases, *ABGC2* was down-regulated and *SLCO1B3* was up-regulated. *ABCG2* was previously found up-regulated in different types of cancer [22,48,49], while the analysis of freely available TCGA data support our qPCR and IHC analysis and show that ABCG2 mRNA levels are lower (z score median −2.5) in endometrioid EC versus control tissue and correlate with significantly longer overall survival of patients (TCGA cohort of 1227 patients with endometrioid EC at The cBioPortal for Cancer Genomics; www.cbioportal.org, accessed on 16 October 2020).

To the best of our knowledge this is the first study that has evaluated expression of E1-S transporters in EC. We used the qPCR approach in model cell lines, HEC-1-A and Ishikawa, and 36 paired samples of EC and adjacent control endometrium, and IHC on 46 EC samples and 26 control samples. The presence and function of the selected transporters differentially expressed at the mRNA level was also investigated by using immunocytochemistry or immunohistochemistry, time-course E1-S transport studies and by evaluating effects of inhibitor CsA, and effects of silencing of specific *SLCO* genes. The cell lines that we used are the best available models of well-differentiated endometrial cancer from premenopausal and postmenopausal patients. The model cell lines were authenticated [50], and the vast majority of experiments were performed within one year of authentication. Antibodies used were appropriately validated [51] by us or other groups [37,38,39]. The data obtained from model cell lines and tissue samples suggest that E1-S transporters ABCG2, OSTβ and OATP1B3 have important roles in pathophysiology of EC. However, further studies with larger sample sizes of clinical tissues are still needed to confirm/elucidate the role of E1-S transporters in postmenopausal EC.

## 4. Materials and Methods

### 4.1. Model Cell Lines

The control cell line HIEEC was obtained from Michael A. Fortier (Laval University, Quebec, QC, Canada) on 4th April 2014 as p14. It was originally generated from a primary culture prepared from an endometrial biopsy taken from a 37-year-old woman with confirmed absence of neoplasia and endometriosis, on day 12 of her menstrual cycle [52]. HIEEC cells were grown in RPMI-1640 medium supplemented with 2 mM L-glutamine and 10% foetal bovine serum (FBS) (all from Sigma-Aldrich, St. Louis, MO, USA). HIEEC cells in passage +7 to +12 were used in this study. Cells in passage +8 were authenticated by STR profiling performed by ATCC on 8 March 2018. The Ishikawa cell line (RRID:CVCL_2529) was originally established from an endometrial adenocarcinoma from a 39-year-old Asian woman, and it was purchased on 18th December 2012 from Sigma-Aldrich (ECACC 99040201; Sigma-Aldrich, St. Louis, MO, USA) as p3. Ishikawa cells were cultured in Eagle’s Minimum Essential Medium supplemented with 2 mM NaHCO_3_ (S3817) and 5% FBS (all from Sigma-Aldrich, St. Louis, MO, USA). The Ishikawa cells in passage +11 to +29 were used in this study. Ishikawa cells *p* + 13 were authenticated by STR profiling performed by ATCC on 22 February 2018. The HEC-1-A cell line (RRID: CVCL_0293) was originally established from an endometrial adenocarcinoma from a 71-year-old patient, and it was purchased from the American Type Culture Collection (ATCC_ HTB-112^TM^) on 31st May 2012 as p125. The growth medium for HEC-1-A cells was McCoy’s 5A medium (Sigma-Aldrich, St. Louis, MO, USA), with 10% FBS. HEC-1-A cells in passage +6 to +18 were used in this study. Cells in passage *p* + 15 were authenticated by STR profiling performed by ATCC on 22nd February, 2018. A human breast adenocarcinoma cell line MCF-7 (RRID:CVCL-0031) was originally established from a 69-year-old patient, and it was purchased on 12th January, 2016 from Sigma-Aldrich (86012803; lot number 14/018, passage number +13; Sigma-Aldrich, St. Louis, MO, USA). MCF-7 cells were grown in Minimum Essential Medium Eagle (56416C) with 10% FBS and 0.01 mg/mL insulin (I9278) (all from Sigma-Aldrich, St. Louis, MO, USA). Cells in passage +15 to +18 were used in this study. Authentication by STR profiling was performed by ECACC on 5 October 2016. All cell lines were cultured in media without antibiotics and were negative for mycoplasma infection, which was periodically tested with the MycoAlert^TM^ mycoplasma detection kit (Lonza, Basel, Switzerland).

### 4.2. Endometrial Tissue

The endometrial cancer tissue samples and paired adjacent control endometrium were obtained from 55 patients undergoing hysterectomies (Table 3). The study was approved by the National Medical Ethics Committee of the Republic of Slovenia (ID 0120-429/2017/8, 5 November 2017) with written informed consent required from all subjects involved. The design of the study followed the principles of the Declaration of Helsinki (Ethical Principles for Medical Research Involving Human Subjects), Oviedo convention (Convention on Human Rights and Biomedicine) and Slovenian Code of Medical Ethics. The patients were all treated at the Department of Gynaecology and Obstetrics at the University Medical Centre Ljubljana, from 2003 to 2010. All the samples were collected and processed according to the approved standard clinical operating procedures. The methods were performed in accordance with the relevant guidelines and regulations.

### 4.3. RNA Isolation and Reverse Transcription

The total RNA from HIEEC, Ishikawa and HEC-1-A cells was isolated using the NucleoSpin RNA kits (Machery-Nagel, GmbH&Co., Düren, Germany) and RNA from tissue samples was isolated using Tri Reagent (Sigma-Aldrich, St. Louis, MO, USA), according to the manufacturer instructions. The RNA samples isolated using Tri reagent were additionally cleaned and residual DNA was removed using RNeasy Mini kits and RNase-Free DNase sets (Qiagen, Düsseldorf, Germany), respectively. The purity and quality of extracted RNA were analysed with the Agilent 2100 Bioanalyzer using the RNA 600 Nanokit (Agilent Technologies Inc., Santa Clara, CA, USA). An average RIN was 9.4 ± 0.86 for RNA samples from cell lines and 7.8 ± 0.80 for RNA samples from tissue, which demonstrated that the RNA was of good quality. Samples of the total RNA were reversely transcribed into cDNA using RT2 First Strand Kit (Qiagen, Hilden, Germany) (for PCR low-density arrays) or SuperScript^®^ VILO™ cDNA Synthesis kit (Invitrogen, Thermo Fisher Scientific, Carlsbad, CA, USA) (for analysis with SYBR Green technology) according to manufacturer instructions. The cDNA samples were stored at −20 °C.

### 4.4. Quantitative PCR

Examination of the expression of genes encoding transporters was first performed in model cell lines and in six pairs of cancer and adjacent control endometrium (samples 8, 16, 19, 24, 33 and 66) using Human Drug Transporters RT^2^ Profiler™ PCR array (PAHS-070Z; SABiosciences, A Qiagen Company). The array enables the evaluation of expression of 84 human drug transporter genes, including 14 E1-S transporters, as well as the five reference genes *ACTB, B2M, GAPDH, HPRT1* and *RPLP0*. Quantification was accomplished with the LightCycler 480 Real-Time PCR system (Roche, Basel, Switzerland) and the RT^2^ SYBR Green Master Mix (Qiagen, Hilden, Germany). The reactions were performed as 384-well PCR arrays (4 × 96 layout) with a reaction volume of 10 μL and the thermocycling parameters recommended by Qiagen (40 cycles). Data analysis was performed using a normalization factor calculated based on the geometric mean of the two most stably expressed reference genes (*ACTB* and *HPRT1*).

The final expression analysis for the 15 genes encoding E1-S transporters was performed using SYBR Green I Master (Roche, Basel, Switzerland) and primers that were designed in our laboratory (Table 4). Quantification was accomplished with the Applied Biosystems^®^ ViiA^TM^ 7 Real-Time PCR System (Thermo Fisher Scientific, Waltham, MA, USA) using the following program: 1 cycle 5 min at 95 °C, 45 cycles 10 s at 95 °C, 10 s at 60 °C and 21 s at 72 °C. All of the samples were run in triplicates, using 0.25 μL cDNA, and the reactions were performed in Applied Biosystems^®^ MicroAmp^®^ Optical 384-well plates (Thermo Fisher Scientific, Waltham, MA, USA), in a reaction volume of 5.0 μL. The PCR amplification efficiency was determined from the slope of the log-linear portion of the calibration curve for each gene investigated, and this was accounted for in the further calculations. For gene expression analysis, the normalization factor for each sample was calculated based on the geometric mean of both of the reference genes (*HPRT1*, *POLR2A*) [53]. Gene expression for each sample was calculated from the crossing-point value (Cq) as *E*^−Cq^, divided by the normalization factor. The relative quantification was performed with the comparative Cq method. The Minimum Information for Publication of Quantitative Real-Time PCR Experiments guidelines were considered in the performance and interpretation of the qPCR reactions [54]. The expression of transporters in cell lines HIEEC, Ishikawa and HEC-1-A was analysed in three independent experiments with 2–3 replicates and in 36 paired EC tissue samples.

### 4.5. Western Blotting

The cell lysates were prepared using RIPA Lysis buffer (EMD Millipore Corporation, Temecula, CA, USA) according to the manufacturer instructions. Total protein concentrations were determined using commercial Bradford reagent (Carl Roth GmbH+ Co. KG, Karlsruhe, Germany) with bovine serum albumin (BSA) as standard using BioTek (Winooski, VT, USA) PowerWave XS Microplate reader.

Protein samples (50 μg) were separated by SDS PAGE on 10% Tris-glycine gels and then the proteins were transferred from the gels to PVDF membranes (Millipore, Billerica, MA, USA) using wet transfer according to the classical protocol using the Bio Rad Mini Trans-Blot^®^ Cell.

For STS detection, the membranes were incubated with 5% non-fat milk overnight and 5% BSA for 2 h, to prevent nonspecific binding. The membranes were then incubated for 2 h with validated primary antibodies anti-STS (1:5000 in TTBS with 5% BSA at 4 °C) [15,37,38], which were kindly provided by prof. dr. Gerhard Schuler (Faculty of Veterinary Medicine, Justus-Leibig-University, Giessen, Germany). The membranes were then incubated for 2 h at 4 °C with the horseradish-peroxidase-conjugated secondary antibodies goat anti-rabbit (111-035-045, 1:5000 in TTBS with 2.5% BSA) (Jackson ImmunoResearch laboratories Inc., West Grove, PA, USA). For GAPDH detection, the membranes were incubated for 2 h with 5% non-fat milk at room temperature and for 1 h with validated primary anti-GAPDH antibodies (Sigma-Aldrich, St. Louis, MO, USA, catalog number: G8795, lot number: 045M4799V, 1:5000 in TTBS with 1% non-fat milk at room temperature). In the following step, the membranes were incubated for 1 h at room temperature with the horseradish-peroxidase-conjugated secondary antibodies goat anti-mouse (115-035-062, 1:5000 in TTBS with 1% nonfat milk) (Jackson ImmunoResearch laboratories Inc., West Grove, PA, USA). The SuperSignal^TM^ West Pico Chemiluminescent Substrate (Thermo Fischer Scientific, Waltham, MA, USA) was used for chemiluminescent detection, with an LAS-4000 CCD camera (Fujifilm, Tokyo, Japan). Differential expression of STS was determined after normalization to GAPDH using ImageJ programme. Protein samples from HIEEC, Ishikawa, HEC-1-A and MCF-7 isolated in four independent experiments were analysed by Western blotting, which was repeated two times.

### 4.6. Immunocytochemistry

Cell lines HEC-1-A and Ishikawa were seeded in 6-well plates containing poly L-lysine coated glass coverslips at 6 × 10^4^ and 5 × 10^4^ cells/well, respectively, and grown for 72 h. For immunostaining, cells were gently washed in cold Dulbecco’s Phosphate Buffered Saline (DPBS, Sigma-Aldrich, St. Louis, MO, USA) followed by incubation in 4% formaldehyde (Thermo Scientific, Waltham, MA, USA) for 20 min and in 0.1% Triton X-100 (Sigma-Aldrich, St. Louis, MO, USA) in DPBS for 10 min. Formaldehyde and Triton X-100 were washed out with 15 min washes in DPBS. Cells were blocked in DPBS containing 5% goat normal serum (Invitrogen, Thermo Fisher Scientific, Carlsbad, CA, USA) for 30 min and then incubated in primary antisera (rabbit anti-SLCO1B3, 1:50 (HPA004943, lot A10598 Sigma-Aldrich, St. Louis, MO, USA); mouse anti-ABCG2, 1:100 (B7059, clone BXP-21, lot GR308999-5 Sigma-Aldrich, St. Louis, MO, USA)) in DPBS containing 1% BSA (Sigma-Aldrich, St. Louis, MO, USA) over night at 4 °C. The following day, cells were washed in DPBS four times, 10 min at room temperature and then incubated with goat anti-rabbit secondary antibody Alexa Fluor 488 conjugates (A-11034, lot 1971418 Invitrogen, Thermo Fisher Scientific, Carlsbad, CA, USA) or goat anti-mouse secondary antibody Alexa Fluor 594 conjugates (A-11032, lot 1420905 Invitrogen, Thermo Fisher Scientific, Carlsbad, CA, USA) diluted 1/200 in DPBS for 90 min at room temperature. After an additional four washes (10 min each) in DPBS, glass coverslips were mounted on microscopic slides using Vectashield antifade mounting medium with DAPI (Vector Laboratories, Burlingame, CA, USA). Immunofluorescence controls were based on replacing the primary antibodies with a normal rabbit or mouse serum (both Invitrogen, Thermo Fisher Scientific, Carlsbad, CA, USA) and on omitting the primary antibodies from control sections.

Immunocytochemical evaluation of OATP1B3 and ABCG2 expression was performed independently three and two times, respectively.

### 4.7. Immunofluorescence Quantification

Digital images of cells were taken under 400-fold magnification using Zeiss Axio Imager 2 microscope (Carl Zeiss Microscopy GmbH, Germany). OATP1B3 immunoreactivity in cells was quantified using a custom software (Surfkvad, by Prof. Dr. Marko Kreft, Institute of Pathophysiology, Faculty of Medicine, University of Ljubljana). For the purpose of analysis with Surfkvad, all digital images were taken with the same illumination settings and prepared accordingly using ImageJ1 [55] as follows: digital images were standardized for illumination, converted to greyscale and were then subjected to threshold conversion using the triangle method in order to selectively identify immunoreactive elements. Black-and-white images were then analysed with Surfkvad software. For each cell line, OATP1B3 immunoreactivity in cells was analysed in total of 30 randomly selected fields (10 fields for each independent experiment) and expressed as immunoreactive area per cell.

### 4.8. E1-S Metabolism Studies

Cell lines Ishikawa and HEC-1-A were seeded in 6-well plates at 1.2 × 10^5^ and 3 × 10^5^ cells/well, respectively, in triplicates. The next day, the full growth medium was replaced with medium without FBS and the cells were treated with 16 nM [^3^H]E1-S (NET203, Perkin Elmer Inc.; Waltham, MA, USA) over the time course of 72 h. The steroids were extracted from the medium at different time points (4, 8, 24, 48, and 72 h) with 100% ethylacetat (medium:ethylacetat = 1 mL:1.5 mL). After 1 min vortexing and centrifugation at 1000× *g* for 2 min, the upper layer with organic phase was transferred into the 2 mL tube. The extraction process was repeated three times in a row. The collected samples were dried in a SpeedVac^TM^ and resuspended in 100 µL of 50% acetonitrile in miliQ water. The samples were analysed by HPLC with an online detector of radioactivity, as previously described [33]. Briefly, the mobile phase was 38% acetonitrile in water, and the flow rate was 0.9 mL/min. The column temperature was 38 °C. Conversion rates were obtained after integration of chromatograms. E1-S metabolism was studied in three independent experiments.

### 4.9. LC-HRMS Assay for Steroid Quantification

Ishikawa and HEC-1-A cells were seeded in 6-well plates at cell densities of 1.75 × 10^5^ cells/well and 3.00 × 10^5^ cells/well, respectively. The next day, when the cells reached 70% confluency, were washed twice with DPBS and treated with different concentrations (10, 100, 500 and 1000 nM) of DHEA-S, DHEA, E1-S (E0251, Sigma-Aldrich Chemie GmbH; Deisenhofen, Germany) or E1, dissolved in DMSO (final concentration, 0.05%), and medium without phenol red and FBS. Cells were treated for 48 h, and then the medium was removed and stored at −80 °C in glass tubes (Wheaton^®^ 6 mL tubes, 986492; VWR, Radnor, PA, USA) until LC-HRMS analysis. For normalization purposes, cells in individual wells were counted using a TC20 Automated Cell Counter (Bio-Rad; Hercules, CA, USA).

The ten predominant metabolites (AD, DHEA, DHEA-S, E1, E1-S, E2, E2-G, E2-S, E3 and T) were then quantified using a selective and sensitive LC-HRMS assay, validated according to the ICH Q2(R1) guidelines as described previously [56]. Briefly, cellular supernatants were thawed at room temperature, 2000 µL thereof were mixed with 20 µL deuterated internal standard solution and precleaned using solid phase extraction on Oasis HLB 1 cc SPE cartridges (30 mg; Waters Corporation, Milford, MA, USA). Before loading the samples onto the columns, the cartridges were preconditioned with 2 × 1.0 mL acetonitrile and 3 × 1.0 mL ammonium acetate buffer (10 mM, pH = 5.0). Samples were then washed using 1 × 1.0 mL ammonium acetate buffer (10 mM, pH = 5.0) and 2 × 1.0 mL acetonitrile/ammonium acetate buffer (10 mM, pH 5.0) 10:90 (*v/v*). Subsequently, all analytes were eluted using 2 × 650 µL acetonitrile/ammonium acetate buffer (10 mM, pH = 5.0) 95:5 (*v*/*v*) and evaporated to dryness. Samples were reconstituted in 270 µL acetonitrile/ammonium acetate buffer (10 mM, pH = 5.0) 25:75 (*v*/*v*) and stored at −80 °C until further LC-HRMS analysis.

LC was performed using an UltiMate 3000 RSLC-series system (Thermo Fisher Scientific, Inc., Waltham, MA, USA) coupled to a maXis HD ESI-Qq-TOF mass spectrometer (Bruker Corporation, Bremen, Germany). For the separation of the analytes, a Phenomenex Luna^®^ 3 µm C18(2) 100 Å LC column (250 × 4.6 mm I.D.; Phenomenex, Inc., Torrance, CA, USA), preceded by a Hypersil^®^ BDS-C18 guard column (5 µm, 10 × 4.6 mm I.D.; Thermo Fisher Scientific, Inc.) was used at a constant temperature of 43 °C and a flow rate of 1.0 mL/min. 100 µL of each sample were injected onto the columns and gradient elution was achieved using aqueous ammonium acetate buffer (10 mM, pH = 5.0) as solvent A, and acetonitrile as solvent B. The gradient ranged as follows: 25% solvent B at 0 min, 56.3% solvent B at 19 min, a washing step at 90% solvent B from 19.5 to 24.0 min, and re-equilibration of the columns with 25% solvent B from 24.5 to 30.5 min. The ESI ion source settings were as follows: Capillary voltage, ±4.5 kV; dry gas flow rate, 8.0 L/min N_2_; nebulizer, 1.0 bar N_2_; and dry temperature, 200 °C. The ion transfer parameters were set to 400 V_pp_ funnel RF and 300 V_pp_ multipole RF, the quadrupole ion energy was 8.0 eV, and the collision cell parameters were as follows: Collision RF, 1100 V_pp_; collision energy, 10.0 eV; transfer time, 38 µs; and prepulse storage, 18 µs. Full-scan mass spectra were recorded over the range of *m*/*z* 150–500, and the lower limits of quantification (LLOQs), defined as the concentrations where the signal to noise ratio (S/N) is ≥9, were determined as follows: AD, 74.9 pg/mL; DHEA, 1904.0 pg/mL; DHEA-S, 8.0 pg/mL; E1, 19.0 pg/mL; E1-S, 4.0 pg/mL; E2, 140.9 pg/mL; E2-G, 12.0 pg/mL; E2-S, 3.4 pg/mL; E3, 28.4 pg/mL and T, 54.1 pg/mL. Coefficients of accuracy and precision were below 1.2% and 2.8% RSD, respectively, and quality control samples, containing each analyte at a concentration of 6-fold, 60-fold or 600-fold of the LLOQs, were analysed in triplicate with each batch to ensure accurate quantification results.

### 4.10. E1-S Transport Experiments

For E1-S transport experiments cell lines Ishikawa and HEC-1-A were seeded in T25 flasks at 1.5 × 10^5^ cells/T25 and 2.0 × 10^5^ cells/T25, respectively, in duplicates. After three days, when the cells reached 60% confluency, the full growth medium was replaced with medium without FBS and the cells were grown for further 24 h. On the day of the transport the cells were washed twice with warm DPBS (S5652; Sigma-Aldrich, St. Louis, MO, USA) and incubated 30 min at 37 °C in transport buffer (125 mM NaCl, 4.8 mM KCl, 1.2 mM CaCl_2_, 1.2 mM KH_2_PO_4_, 12 mM MgSO_4,_ 25 mM MES, and 5.6 mM glucose, with the pH adjusted to 5.5 [57]). After 30 min incubation period the steroid transport was initiated by replacing the transport buffer with warm (37 °C) or cold (4 °C) transport buffer containing 16 nM [^3^H]E1-S (NET203250UC, Perkin Elmer Inc., MA, USA), and the cells were incubated for 1 s, 1 min, 2.5 min, 5 min, 10 min, 15 min and 30 min at 37 °C or 4 °C. For determination of the effect of inhibitor cyclosporine A (CsA) on E1-S cellular uptake, the cells were initially incubated for 30 min in transport buffer containing different concentrations (0.01 µM, 0.1 µM, 10 µM) of CsA (SML1018, C1832, Sigma-Aldrich, St. Louis, MO, USA) in DMSO, final concentration up to 1.2%, and then the steroid transport was initiated by adding [^3^H]E1-S to final concentration 16 nM. After incubation, the medium was collected, and the uptake was stopped with 2 mL of ice-cold DPBS. After series of washing (five times) with DPBS, the cells were solubilized in 300 µL of 0.1% Triton X-100, shaken for 30 min at 200 rot./min at 4 °C and frozen at −80 °C overnight. The next day the cell suspensions were collected and centrifuged at 12.000 rpm for 15 min at 4 °C. Finally, 250 µL of cell lysate was mixed with 1.5 mL of scintillation fluid (Quickszint Flow 302, Zinnsser Analytic, Frankfurt, Germany) and the radioactivity was measured with MicroBeta^®^ TriLux 1450 detector (PerkinElmer, CT, USA). Molarity of [^3^H]E1-S in individual samples was calculated as the quotient of disintegrations per minute (DPM) and specific isotope activity (49.19 Ci/mmol) with conversion 1 Ci = 2.22 × 10^12^ DPM. The results were normalized to total protein concentration determined using BCA (Pierce™BCA Protein Assay Kit, Thermo Scientific) according to manufacturer instructions. Time-dependence of E1-S transport was studied in three independent experiments. Effects of CsA on E1-S transport were evaluated in two to four independent experiments.

### 4.11. Gene Silencing Using Small-Interfering (si)RNAs

For siRNA-mediated gene silencing cell line HEC-1-A was seeded in 6-well plates at cell density of 1.2 × 10^5^ cells/well. After 24 h, when the cells reached 40% confluency, transient transfection was performed using individual (30 pmol) or a combination (20 pmol each) of predesigned Silencer Select siRNAs (4392420, ThermoFisher Scientific, Waltham, MA, USA) targeting genes *SLCO1B3* (s26263, lot ASO2BB05)*, SLCO1B1* (s20805, lot ASO2F1WJ) and/or *SLCO2B1* (s22292, lot ASO2F1WK) using PepMute™ siRNA Transfection Reagent (SL100566, SignaGen, Frederick, MD, USA) according to the manufacturer instructions. As a negative or positive control, Silencer Select Negative control #1 (4390843, ThermoFisher Scientific) or Silencer Select GAPDH siRNA (4390849, ThermoFisher Scientific) were used, respectively. Transfection was performed in full growth medium which was then replaced with fresh one 24 h after transfection. The cells were grown for a further 24 h, when combination of *SLCO1B3, SLCO1B1* and *SLCO2B1* was targeted, or for further 48 h when only *SLCO1B3* or *SLCO2B1* were targeted, and then 2-min [^3^H]E1-S uptake was performed at 37 °C using similar protocol as described in section “E1-S transport experiments”.

### 4.12. Immunohistochemistry

Tissue microarrays (TMA) included 3 mm cores of tumour and 2 mm cores of adjacent control endometrium from formalin-fixed, paraffin embedded tissues. In total, there were 46 tumour samples and 26 control samples of which 20 samples were paired. Immunohistochemical stainings for OSTβ, ABCG2 and OATP1B3 were performed on a fully automated Ventana BenchMark ULTRA System (Ventana Medical Systems, Inc., Tuscon, AZ, USA). In the pretreatment process, all of the protocols included Cell Conditioning Solution 1 (37 °C; Tris-based buffer with pH 8.5) and an incubation step with H_2_O_2_ to block endogenous peroxidase. All of the technical conditions of the protocols that were used for the staining are included in Table S1. The anti-OSTβ (1:100/TPBS, 1 h, HPA008533, lot A105958; Sigma Aldrich, St. Louis, MO, USA,), anti-ABCG2 (1:50/TPBS, 1 h, ab3380, lot GR308999-5, clone number BXP-21; Abcam, Cambridge, UK) and anti-OATP1B3 (1:50/TBS, 2 h, HPA004943, lot E104368; Sigma Aldrich, St. Louis, MO, USA) were used and applied manually during the immunohistochemical process. These antibodies were validated using appropriate negative and positive controls (Figure S2). For IHC with anti-OSTβ and anti-ABCG2 antibodies [39], samples of normal colonic mucosa were used as positive controls, and for anti-OATP1B3 we used normal liver tissue as a positive control (Figure 6). Staining was assessed according to the percentage (P) of positive stained cells in the endometrial glands and staining intensity (I), scored as: 0, negative, 1, weak positive reaction; 2, strong positive reaction). The score was calculated by multiplying the percentage with the intensity (scoring = P × I; values ranging from 0 to maximum of 200). If the sample was duplicated the average staining score was used for statistical analysis. Immunohistochemical staining was evaluated by a pathologist (S.F-G.).

### 4.13. Statistical Analysis

Statistical evaluation was carried out using GraphPad Prism Software for Windows, version 8.0.0 (San Diego, CA, USA). Experiments were repeated two to three times in two to four technical replicates. In EC tissue gene expression was evaluated in 36 paired samples. Immunohistochemistry was performed on 56 tissue samples, where 20 were paired (pairs of EC and adjacent control tissue of the same patient). Statistical analysis was performed using the Kruskal–Wallis test with Dunn’s multiple comparison test correction (expression of ‘transporter genes’ in cell lines, siRNA silencing of *SLCO1B3* and *SLCO2B1*), Wilcoxon matched pairs with Bonferroni’s multiple comparison test (expression of ‘transporter genes’ in paired EC tissue samples), one-way analysis of variance (ANOVA) with Bonferroni’s multiple comparison tTest (Western blotting), two-way ANOVA (analysis of association between transporters’ expression and histopathological and clinical data), Mann–Whitney U test (immunocytochemistry, [^3^H]E1-S transport and metabolism studies), paired *t*-test (immunohistochemistry). Differences of *p* < 0.05 were considered statistically significant. Unless stated otherwise, all data are shown as mean ± SD.

## 5. Conclusions

In a model cell line of moderately/well differentiated EC from postmenopausal patient (HEC-1-A), E1-S metabolism was enhanced [33] as compared to a model cell line of well differentiated EC from a premenopausal patient (Ishikawa). These results can be explained by down-regulation and low protein levels of the efflux transporter ABCG2, higher levels of OATPs, especially up-regulation and increased protein levels of *SLCO1B3* (OATP1B3), as confirmed by immunocitochemistry, differences in CsA inhibition profiles and siRNA-mediated gene-silencing, and also by increased protein levels of STS in HEC-1-A versus Ishikawa. In EC tissue, we previously reported that increased levels of E2 are formed via the sulfatase pathway [15]. Current data show that in EC patients, down-regulation and low protein levels of E1-S efflux transporters *ABCG2* (ABCG2) and *SLC51B* (OSTβ) may affect cellular E1-S levels and thus also E2 formation. Taken together, our data indicate that E1-S transport has probably a more important role in postmenopausal women and EC patients with low-grade tumours, FIGO IA stage, with < 50% invasion into myometrium and without LVI.

## Figures and Tables

**Table 1:** E1-S uptake and efflux transporters from the *ABC* and *SLC* gene families.

Genes	Proteins	K_M_ (μM) *
*ABC*		
*ABCC1*	ABCC1/MRP1	0.7–4.2
*ABCC4*	ABCC4/MRP4	nd
*ABCC11*	ABCC11/MRP8	nd
*ABCG2*	ABCG2/BCRP	6.8–16.6
*SLC21 = SLCO*		
*SLCO1A2*	OATP1A2	7–59
*SLCO1B1*	OATP1B1	0.1–45
*SLCO1B3*	OATP1B3	5–58
*SLCO2B1*	OATP2B1	1.6–21
*SLCO1C1*	OATP1C1	nd
*SLCO3A1*	OATP3A1	nd
*SLCO4A1*	OATP4A1	nd
*SLCO4C1*	OATP4C1	27
*SLC22*		
*SLC22A6*	OAT1	nd
*SLC22A7*	OAT2	nd
*SLC22A8*	OAT3	2.2–21
*SLC22A11*	OAT4	1–22
*SLC22A9*	OAT7	8.7
*SLC10*		
*SLC10A6*	SOAT	nd
*SLC51*		
*SLC51A*	OSTα	320
*SLC51B*	OSTβ	320

* Data from review papers [18,19,22,24]. Where known, K_M_ values for transport of E1-S are given.

**Table 2:** Data on the antibodies used for Western blotting or immunohistochemistry (IHC).

Antigen, Type of Antibodies	Manufacturer, Catalogue ID, Batch ID, Name of the Source	Antigen Sequence	Species Raised, Monoclonal, Polyclonal, Antigen Purified	Positive and Negative Controls	Method Where Antibodies Were Used
anti-STS	Provided by Dr. Schuler [37,38].	whole protein	rabbit, polyclonal	HepG2 (positive), MCF-7 (negative)	Western blot
anti-OSTβ rabbit, polyclonal	HPA008533, lot A105958; Sigma Aldrich, St. Louis, MO, USA	VLHLDEAKDHNSLNNLRETLLSEKPNLAQVELELKERDVLSVFLPDV	rabbit, polyclonal, affinity purified	colon (positive) liver (negative)	IHC
anti-ABCG2	ab3380, lot GR308999-5, clone number BXP-21; Abcam, Cambridge, UK	126 amino acids, 271–396 GeneBank accession #AF098951]	mouse monoclonal	colon (positive) liver (negative) [39]	IHC
anti-OATP1B3	HPA004943, lot E104368; Sigma Aldrich, St. Louis, MO, USA	QGKDTKASDNERKVMDEANLEFLNNGEHFVPSAGTDSKTCNLDMQDNAAAG	rabbit, polyclonal, affinity purified	WB: HEPG2 (positive) MCF7 (negative) IHC: liver tissue (positive), cardiac muscle (negative)	IHC

**Table 3:** Detailed information about patients included in this study.

Sample	Age	Menopausal Status	Histological Type/Grade	Limfovascular Invasion (LVI)	FIGO Stage	Depth of Miometrial Invasion	Gradus HG/LG	Gene Expression qPCR	Protein Levels IHC
2	52	NA	G1 *	yes	IA	<50%	L	no	yes ^§^
3	65	postmenopausal	G1 *	no	IA	<50%	L	no	yes
5	39	premenopausal	dedifferentiated	yes	IB	>50%	H	yes	no
6	76	postmenopausal	serous	yes	IIIC	>50%	H	no	yes ^§^
7	50	premenopausal	G1 *	no	IB	Not present	L	yes	yes ^§^
8	83	postmenopausal	dedifferentiated	no	IB	>50%	H	yes	no
9	41	premenopausal	G1 *	no	IA	<50%	L	yes	yes
10	53	postmenopausal	G1 *	no	IA	not present	L	yes	yes
11	60	postmenopausal	G2 *	yes	IB	>50%	L	no	yes ^§^
13	64	postmenopausal	G1 *	NA	IV	<50%	L	yes	yes ^§^
14	73	postmenopausal	G1 *	no	IB	>50%	L	yes	yes ^§^
15	64	postmenopausal	G2 *	yes	IB	>50%	L	no	yes ^§^
16	69	postmenopausal	G1 *	no	IA	<50%	L	yes	yes ^§^
18	79	postmenopausal	G1 *	no	IB	>50%	L	yes	yes ^§^
19	74	postmenopausal	G1 *	no	IA	<50%	L	yes	yes
20	76	postmenopausal	G1 *	no	IA	<50%	L	yes	yes ^§^
21	53	premenopausal	G2 *	no	IA	not present	L	yes	yes ^§^
22	36	premenopausal	G1 *	no	IA	not present	L	no	yes ^§^
23	45	premenopausal	G1 *	no	IA	not present	L	yes	yes ^§^
24	69	postmenopausal	G2 *	yes	IB	>50%	L	yes	yes ^§^
25	54	premenopausal	G3 *	no	IA	<50%	H	yes	yes
26	72	postmenopausal	G1 *	no	IA	<50%	L	yes	yes
30	54	premenopausal	G1 *	no	IA	not present	L	yes	yes ^§^
31	69	postmenopausal	G3 *	yes	IB	>50%	H	no	yes ^§^
33	77	postmenopausal	G3 *	no	IB	>50%	H	yes	yes ^§^
34	57	postmenopausal	mucinous, G1 *	no	IA	<50%	L	yes	yes ^§^
35	61	postmenopausal	G1 *	no	IA	not present	L	no	yes
38	78	postmenopausal	G2 *	yes	IA	<50%	L	no	yes
39	63	postmenopausal	G1 *	yes	IA	<50%	L	no	yes ^§^
40	71	postmenopausal	serous	no	IA	<50%	H	yes	yes ^§^
44	73	postmenopausal	serous	yes	IB	>50%	H	yes	yes ^§^
46	50	premenopausal	G2 *	no	IIIA	<50%	L	no	yes
47	27	premenopausal	dedifferentiated	no	IA	<50%	H	yes	yes
48	59	postmenopausal	serous	yes	IB	>50%	H	no	yes ^§^
49	70	postmenopausal	G1 *	no	IA	<50%	L	yes	yes
50	73	postmenopausal	G1 *	no	IA	<50%	L	yes	yes ^§^
51	75	postmenopausal	G2 *	yes	IA	<50%	L	yes	yes ^§^
52	75	postmenopausal	G2 *	yes	IA	<50%	L	yes	yes ^§^
53	50	postmenopausal	G3 *	yes	IA	<50%	H	yes	yes ^§^
54	71	postmenopausal	G1 *	no	IA	<50%	L	yes	yes
55	75	postmenopausal	serous	yes	IIIC	>50%	H	no	yes ^§^
56	55	postmenopausal	G1 *	no	IA	not present	L	yes	yes
57	43	premenopausal	G1 *	no	IA	not present	L	yes	yes
58	68	postmenopausal	G2 *	no	IA	<50%	L	no	yes ^§^
60	55	postmenopausal	G1 *	no	IA	not present	L	no	yes ^§^
61	83	postmenopausal	G1 *	no	IA	not present	L	no	yes
62	59	postmenopausal	G1 *	no	IA	not present	L	yes	yes
63	66	postmenopausal	G1 *	no	IA	<50%	L	yes	yes
64	66	postmenopausal	G1 *	no	IA	<50%	L	no	yes
65	80	postmenopausal	carcinosarcoma	yes	IB	>50%	H	yes	yes ^§^
66	72	postmenopausal	G1 *	no	IA	<50%	L	yes	yes ^§^
68	45	premenopausal	G1 *	no	II	<50%	L	yes	yes ^§^
69	72	postmenopausal	G1 *	no	IA	<50%	L	no	yes ^§^
70	55	postmenopausal	G3 *	NA	IB	>50%	H	no	yes
71	48	premenopausal	serous	no	IA	<50%	H	yes	yes

^§^ samples where only tumour or only control tissue was included in TMA (i.e., not paired samples), * endometrioid.

**Table 4:** Sequences of the primers used for amplification of genes of interest.

Gene	Forward Primers	Reverse Primers
*ABCC1*	5′-GGACTCAGGAGCACACGAAA-3′	5′-ACGGCGATCCCTTGTGAAAT-3′
*ABCC4*	5′-AACTGCAACTTTCACGGATG-3′	5′-AATGACTTTTCCCAGGCGTA-3′
*ABCG2*	5′-GGGTTTGGAACTGTGGGTAG-3′	5′-AGATGATTCTGACGCACACC-3′
*SLC10A6*	5′-TATGACAACCTGTTCCACCG-3′	5′-GAATGGTCAGGCACACAAGG-3′
*SLC22A11*	5′-CTCACCTTCATCCTCCCCTG-3′	5′-CCATTGTCCAGCATGTGTGT-3′
*SLC51A*	5′-GCCCTTTCCAATACGCCTTC-3′	5′-TCTGCTGGGTCATAGATGCC-3′
*SLC51B*	5′-GTGCTGTCAGTTTTCCTTCCG-3′	5′-TCATGTGTCTGGCTTAGGATGG-3′
*SLCO1A2*	5′-GTTGGCATCATTCTGTGCAAATGTT-3′	5′-AACGAGTGTCAGTGGGAGTTATGAT-3′
*SLCO1B3*	5′-TCCAGTCATTGGCTTTGCAC-3′	5′-TCCAACCCAACGAGAGTCCT-3′
*SLCO1C1*	5′-CACACAGACTACCAAACACCC-3′	5′-TCACCATGCCGAACAGAGAA-3′
*SLCO2B1*	5′-AGAGCCCTGTGTTCCATTCT-3′	5′-CTCTTGCTCCAGAAATGGCC-3′
*SLCO3A1*	5′-CTACGACAATGTGGTCTAC-3′	5′-TTTTGATGTAGCGTTTATAG-3′
*SLCO4C1*	5′-CCAGGAGCCCCAGAAGTC-3′	5′-AACTCGGACAGCGACAGTG-3′
*SLCO4A1*	5′-ATGCACCAGTTGAAGGACAG-3′	5′-AACAAGGTGGCAGCTTCTGAG-3′
*SLCO1B1*	5′-CAAATTCTCATGTTTTACTG-3′	5′-GATTATTTCCATCATAGGTC-3′

## Data Availability

The data presented in this study are available in Supplementary Material.

## Supplementary Materials

Supplementary Materials can be found at https://www.mdpi.com/article/10.3390/ijms22083819/s1, Figure S1: Uncropped gels and blots in Western blot detection of STS and GAPDH, Figure S2: Positive and negative controls for anti-ABCG2, anti-OSTβ, and anti-OATP1B3 antibodies, used in immunohistochemical staining, Table S1: Technical conditions for immunohistochemical staining. Table S2: Normalized expression of evaluated genes in model cell lines and tissue samples evaluated using qPCR, Table S3: Results of DHEA-S, DHEA, E1-S and E1 metabolism in model cell lines of EC evaluated using LC-HRMS, Table S4: Results of immunohistochemistry presented as scores.
