# Supplementary material for: Altered Profile of E1-S Transporters in Endometrial Cancer: Lower Protein Levels of ABCG2 and OSTβ and Up-Regulation of SLCO1B3 Expression
Source: Int J Mol Sci. 2021 Apr 7;22(8):3819. doi: 10.3390/ijms22083819 (PMC8067723; doi:10.3390/ijms22083819)
Supplement: Supplementary file 1 [file ijms-22-03819-s001.pdf]

Supplementary Materials: Altered Profile of E1-S Transporters in Endometrial Cancer: Lower Protein Levels of ABCG2 and OSTβ and Up-Regulation of *SLCO1B3* Expression

Renata Pavlič, Suzana Vidic, Maja Anko, Tamara Knific, Tomaž Büdefeld, Kristina Marton, Maša Sinreih, Stefan Poschner, Walter Jäger, Snježana Frković-Grazio and Tea Lanišnik Rižner

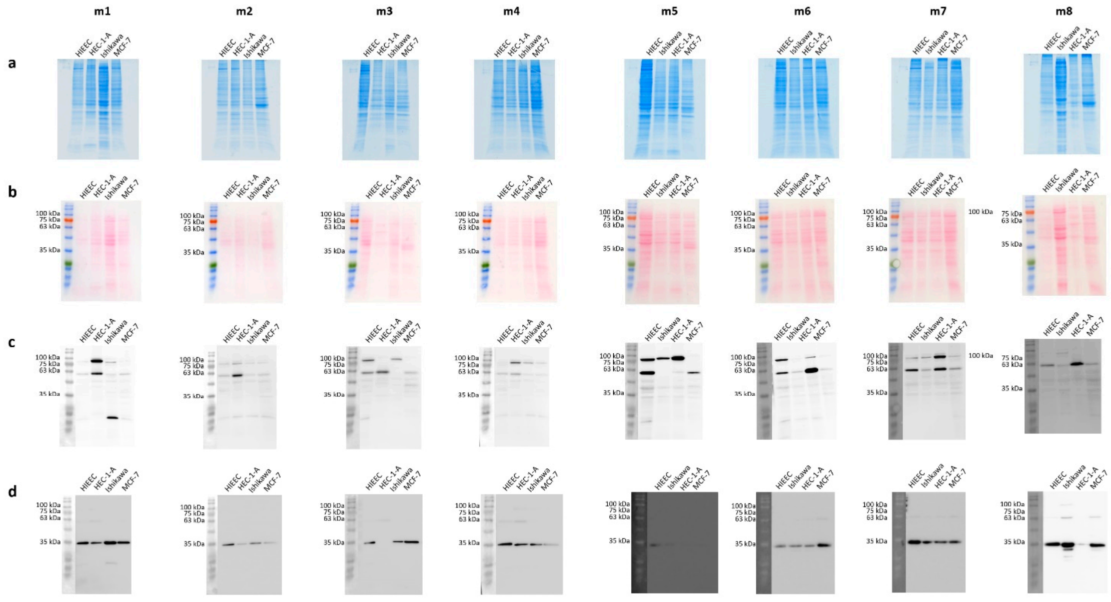

e

Protein	Expected band position	m1 HIEEC	m1 HEC-1-A	m1 Ishikawa	m1 MCF-7	m2 HIEEC	m2 HEC-1-A	m2 Ishikawa	m2 MCF-7	m3 HIEEC	m3 HEC-1-A	m3 Ishikawa	m3 MCF-7	m4 HIEEC	m4 HEC-1-A	m4 Ishikawa	m4 MCF-7
		Densitometric readings acquired with Image J (area)															
Post-translationally modified STS	at approx. 100 kDa	630.557	26183.693	3589.660	255.021	3355.882	5395.267	1993.033	1358.719	13883.915	467.506	7778.874	344.263	1446.749	14513.844	5453.560	1804.326
STS	at approx. 63 kDa	1890.861	14628.238	1110.234	2080.154	5184.267	23825.158	1889.619	2975.589	11072.187	18591.329	0.000	4369.146	3424.832	5994.560	1046.234	1184.477
GAPDH	at approx. 35 kDa	16537.066	10614.752	23807.501	17465.238	15061.673	4013.832	10436.359	4382.660	5261.024	0.000	4479.368	12885.602	15.404.794	13180.794	9171.409	3806.125

Protein	Expected band position	m5 HIEEC	m5 Ishikawa	m5 HEC-1-A	m5 MCF-7	m6 HIEEC	m6 Ishikawa	m6 HEC-1-A	m6 MCF-7	m7 HIEEC	m7 Ishikawa	m7 HEC-1-A	m7 MCF-7	m8 HIEEC	m8 Ishikawa	m8 HEC-1-A	m8 MCF-7
		Densitometric readings acquired with Image J (area)															
Post-translationally modified STS	at approx. 100 kDa	24172.844	17172.167	32110.643	232.607	14184.217	269.192	3495.861	359.435	4727.832	4176.175	25427.765	1620.376	597.849	1269.648	0.000	179.778
STS	at approx. 63 kDa	28857.208	255.192	929.991	9824.388	11753.317	2293.276	34246.037	1683.912	19335.480	3835.660	23165.430	6318.267	7918.167	4181.196	26435.179	4152.782
GAPDH	at approx. 35 kDa	10131.903	2794.983	691.355	698.577	4860,246	58732.614	5433.196	12387.631	15023.581	10219.317	8672.953	9038.196	19473.844	27079.635	6871.581	18696.744

Figure S1. Uncropped gels and blots in Western blot detection of STS and GAPDH. a) Gels after staining with Coomassie brilliant blue, b) full-length membranes after staining with Ponceau S, c) full-length membranes after chemiluminescent detection of STS, d) full-length membranes after chemiluminescent detection of GAPDH, e) densitometric readings for quantification acquired with programme Image J. Membrane 7 (m7) was chosen as representative membrane of STS levels in investigated cell lines.

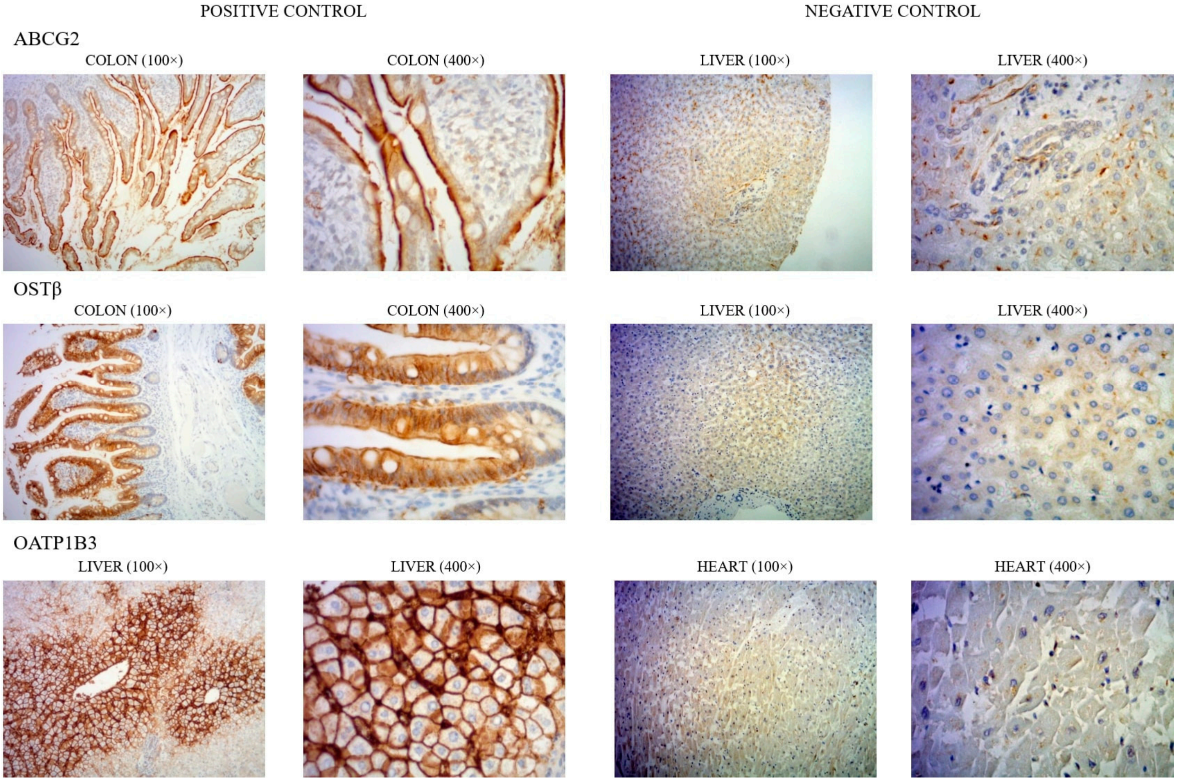

Figure S2. Positive and negative controls for anti-ABCG2, anti-OSTβ, and anti-OATP1B3 antibodies, used in immunohistochemical staining.

Table S1. Technical conditions for immunohistochemical staining.

Primary antibody	Pre-treatment	Pre-treatment (min)	Primary antibody dilution	Primary antibody incubation (min)	Antibody diluent	Primary antibody incubation temperature (°C)	Detection kit	Thickness
ABCG2	CC1	48	1:50	60	TTBS	37	OptiView	4 µm
OSTβ	CC1	48	1:100	60	TTBS	37	OptiView	4 µm
OATP1B3	CC1	72	1:50	120	TBS	37	OptiView	4 µm

IHC was performed on the Ventana BenchMark ULTRA platform. CC1 – Cell Conditioning Solution 1 (Tris-based buffer, pH 8.5), TBS – Tris-Buffered Saline, TTBS – Tween-Tris-Buffered Saline.

Table S2. Normalized expression of evaluated genes in model cell lines and tissue samples evaluated using qPCR.

	**HIEEC**		**Ishikawa**		**HEC-1-A**		**Normal tissue**		**Tumor tissue**	
gene	**mean**	**SD**	**mean**	**SD**	**mean**	**SD**	**mean**	**SD**	**mean**	**SD**
ABCC1	212347.91	120167.83	793260.86	871703.41	287172.96	185450.98	171007.00	105071.00	268428.00	239633.00
ABCC4	17563.24	7331.72	93064.76	106863.99	252419.40	231728.27	64531.10	74076.40	59001.10	47012.40
ABCG2	41691.98	15412.68	26622.47	25065.35	1329.01	1271.83	905358.00	779787.00	299870.00	780028.00
SLC10A6	524.56	137.10	4287.98	4711.50	3841.04	3018.53	20421.20	20447.50	32124.90	110341.00
SLC22A11	106.17	70.20	135.98	85.85	976.17	954.03	11375.70	9168.96	7678.84	7745.00
SLC51A	14797.83	8470.23	207015.19	295527.05	92745.15	99959.00	44121.50	44178.70	59825.20	55194.40
SLC51B	1439.37	536.86	61868.48	96982.06	9572.80	11705.51	180466.00	134256.00	85590.40	107595.00
SLCO1A2	338.55	202.35	11891.72	20194.06	107.75	135.02	6724.91	16820.90	24512.90	87127.70
SLCO1B1	10.82	6.81	397.36	298.16	6500.27	7250.01	88513.40	205123.00	85141.20	179207.00
SLCO1B3	410.02	394.39	13556.37	22706.14	8212224.39	7316381.75	353300.00	751143.00	3020220.00	13262300.00
SLCO1C1	32.90	20.13	1777.63	3210.93	683.96	1307.26	3509.41	2818.91	3803.15	4128.28
SLCO2B1	1407.00	389.01	1010.77	1238.63	142565.28	100526.24	392533.00	285953.00	358978.00	486884.00
SLCO3A1	925.72	815.48	49.26	85.39	278.00	220.62	9883.77	14303.70	8493.11	16459.40
SLCO4A1	8896.66	5529.25	11596.36	12879.74	346579.01	249855.69	281831.00	366875.00	286660.00	516142.00
SLCO4C1	3466.15	852.02	408303.85	354990.02	852.59	971.24	14510.30	15098.10	26496.60	50860.70

Table S3. Results of DHEA-S, DHEA, E1-S and E1 metabolism in model cell lines of EC evaluated using LC-HRMS.

	Metabolite (calculated concentration)									
Sample ID	DHEA	DHEA-S	AD	T	E1	E1-S	E2	E2-S	E2-G	E3
Ishikawa control	n.c.	n.c.	n.c.	n.c.	n.c.	n.c.	n.c.	n.c.	n.c.	n.c.
Ishikawa control	n.c.	n.c.	n.c.	n.c.	n.c.	n.c.	n.c.	n.c.	n.c.	n.c.
Ishikawa 10nM DHEA	9.197 nM	30.749 fmol/Mio/h	n.c.	n.c.	n.c.	n.c.	n.c.	n.c.	n.c.	n.c.
Ishikawa 10nM DHEA	9.213 nM	17.664 fmol/Mio/h	n.c.	n.c.	n.c.	1.264 fmol/Mio/h	n.c.	n.c.	n.c.	n.c.
Ishikawa 100nM DHEA	93.775 nM	195.921 fmol/Mio/h	n.c.	n.c.	n.c.	n.c.	n.c.	n.c.	n.c.	n.c.
Ishikawa 100nM DHEA	91.836 nM	150.140 fmol/Mio/h	n.c.	n.c.	n.c.	n.c.	n.c.	n.c.	n.c.	n.c.
Ishikawa 500nM DHEA	388.361 nM	481.770 fmol/Mio/h	23.700 fmol/Mio/h	n.c.	n.c.	2.040 fmol/Mio/h	n.c.	n.c.	n.c.	n.c.
Ishikawa 500nM DHEA	438.961 nM	428.753 fmol/Mio/h	27.026 fmol/Mio/h	n.c.	n.c.	2.591 fmol/Mio/h	n.c.	n.c.	n.c.	n.c.
Ishikawa 1000nM DHEA	780.560 nM	945.828 fmol/Mio/h	78.053 fmol/Mio/h	n.c.	n.c.	2.322 fmol/Mio/h	n.c.	n.c.	n.c.	n.c.
Ishikawa 1000nM DHEA	755.463 nM	811.222 fmol/Mio/h	46.373 fmol/Mio/h	n.c.	n.c.	1.416 fmol/Mio/h	n.c.	n.c.	n.c.	n.c.
Ishikawa 10nM E1	n.c.	4.770 fmol/Mio/h	n.c.	n.c.	3.026 nM	79.650 fmol/Mio/h	78.437 fmol/Mio/h	32.044 fmol/Mio/h	n.c.	n.c.
Ishikawa 10nM E1	n.c.	3.084 fmol/Mio/h	n.c.	n.c.	3.043 nM	85.446 fmol/Mio/h	85.344 fmol/Mio/h	32.635 fmol/Mio/h	n.c.	n.c.
Ishikawa 100nM E1	n.c.	3.678 fmol/Mio/h	n.c.	n.c.	51.361 nM	107.343 fmol/Mio/h	1470.020 fmol/Mio/h	69.065 fmol/Mio/h	n.c.	n.c.
Ishikawa 100nM E1	n.c.	1.960 fmol/Mio/h	n.c.	n.c.	56.220 nM	84.602 fmol/Mio/h	896.104 fmol/Mio/h	49.760 fmol/Mio/h	n.c.	n.c.
Ishikawa 500nM E1	n.c.	7.584 fmol/Mio/h	n.c.	n.c.	386.867 nM	328.617 fmol/Mio/h	3023.847 fmol/Mio/h	226.756 fmol/Mio/h	n.c.	n.c.
Ishikawa 500nM E1	n.c.	66.104 fmol/Mio/h	n.c.	n.c.	370.105 nM	451.509 fmol/Mio/h	4056.246 fmol/Mio/h	296.022 fmol/Mio/h	n.c.	n.c.
Ishikawa 1000nM E1	n.c.	n.c.	n.c.	n.c.	595.627 nM	896.478 fmol/Mio/h	18305.047 fmol/Mio/h	461.948 fmol/Mio/h	n.c.	n.c.
Ishikawa 1000nM E1	n.c.	n.c.	n.c.	n.c.	615.821 nM	637.871 fmol/Mio/h	12119.243 fmol/Mio/h	294.448 fmol/Mio/h	n.c.	n.c.
Ishikawa control	n.c.	n.c.	n.c.	n.c.	n.c.	n.c.	n.c.	n.c.	n.c.	n.c.
Ishikawa control	n.c.	n.c.	n.c.	n.c.	n.c.	n.c.	n.c.	n.c.	n.c.	n.c.
Ishikawa 10nM DHEA-S	n.c.	6.580 nM	n.c.	n.c.	n.c.	n.c.	n.c.	n.c.	n.c.	n.c.
Ishikawa 10nM DHEA-S	n.c.	6.308 nM	n.c.	n.c.	n.c.	n.c.	n.c.	n.c.	n.c.	n.c.
Ishikawa 100nM DHEA-S	n.c.	61.750 nM	n.c.	n.c.	n.c.	n.c.	n.c.	n.c.	n.c.	n.c.
Ishikawa 100nM DHEA-S	n.c.	61.751 nM	n.c.	n.c.	n.c.	n.c.	n.c.	n.c.	n.c.	n.c.
Ishikawa 500nM DHEA-S	n.c.	491.766 nM	n.c.	n.c.	n.c.	n.c.	n.c.	n.c.	n.c.	n.c.
Ishikawa 500nM DHEA-S	n.c.	461.321 nM	n.c.	n.c.	n.c.	n.c.	n.c.	n.c.	n.c.	n.c.
Ishikawa 1000nM DHEA-S	n.c.	919.895 nM	n.c.	n.c.	n.c.	n.c.	n.c.	n.c.	n.c.	n.c.
Ishikawa 1000nM DHEA-S	n.c.	933.723 nM	n.c.	n.c.	n.c.	n.c.	n.c.	n.c.	n.c.	n.c.
Ishikawa 10nM E1-S	n.c.	n.c.	n.c.	n.c.	11.713 fmol/Mio/h	7.950 nM	n.c.	0.972 fmol/Mio/h	n.c.	n.c.
Ishikawa 10nM E1-S	n.c.	n.c.	n.c.	n.c.	30.588 fmol/Mio/h	8.989 nM	n.c.	2.493 fmol/Mio/h	n.c.	n.c.
Ishikawa 100nM E1-S	n.c.	0.024 nM	n.c.	n.c.	128.292 fmol/Mio/h	80.673 nM	n.c.	8.949 fmol/Mio/h	n.c.	n.c.
Ishikawa 100nM E1-S	n.c.	0.030 nM	n.c.	n.c.	250.570 fmol/Mio/h	82.692 nM	n.c.	12.171 fmol/Mio/h	n.c.	n.c.
Ishikawa 500nM E1-S	n.c.	n.c.	n.c.	n.c.	1016.009 fmol/Mio/h	456.395 nM	134.201 fmol/Mio/h	21.079 fmol/Mio/h	n.c.	n.c.
Ishikawa 500nM E1-S	n.c.	n.c.	n.c.	n.c.	2013.594 fmol/Mio/h	405.687 nM	342.191 fmol/Mio/h	20.579 fmol/Mio/h	n.c.	n.c.
Ishikawa 1000nM E1-S	n.c.	n.c.	n.c.	n.c.	2055.953 fmol/Mio/h	894.851 nM	318.671 fmol/Mio/h	31.475 fmol/Mio/h	n.c.	n.c.
Ishikawa 1000nM E1-S	n.c.	n.c.	n.c.	n.c.	4357.706 fmol/Mio/h	867.621 nM	705.346 fmol/Mio/h	31.994 fmol/Mio/h	n.c.	n.c.
HEC-1-A control	n.c.	n.c.	n.c.	n.c.	n.c.	n.c.	n.c.	n.c.	n.c.	n.c.
HEC-1-A control	n.c.	n.c.	n.c.	n.c.	n.c.	n.c.	n.c.	n.c.	n.c.	n.c.
HEC-1-A 10nM DHEA	6.705 nM	48.277 fmol/Mio/h	n.c.	n.c.	n.c.	n.c.	n.c.	n.c.	n.c.	n.c.
HEC-1-A 10nM DHEA	7.187 nM	64.807 fmol/Mio/h	n.c.	n.c.	n.c.	n.c.	n.c.	n.c.	n.c.	n.c.
HEC-1-A 100nM DHEA	72.971 nM	308.462 fmol/Mio/h	n.c.	n.c.	n.c.	n.c.	n.c.	n.c.	n.c.	n.c.
HEC-1-A 100nM DHEA	80.207 nM	625.002 fmol/Mio/h	n.c.	n.c.	n.c.	n.c.	n.c.	n.c.	n.c.	n.c.

HEC-1-A 500nM DHEA	335.670 nM	6016.566 fmol/Mio/h	59.537 fmol/Mio/h	n.c.	n.c.	n.c.	n.c.	n.c.	n.c.	n.c.
HEC-1-A 500nM DHEA	299.632 nM	3502.040 fmol/Mio/h	31.928 fmol/Mio/h	n.c.	n.c.	n.c.	n.c.	n.c.	n.c.	n.c.
HEC-1-A 1000nM DHEA	480.195 nM	4816.805 fmol/Mio/h	66.456 fmol/Mio/h	n.c.	n.c.	0.707 fmol/Mio/h	n.c.	0.673 fmol/Mio/h	n.c.	n.c.
HEC-1-A 1000nM DHEA	527.306 nM	5063.847 fmol/Mio/h	58.499 fmol/Mio/h	n.c.	n.c.	n.c.	n.c.	0.966 fmol/Mio/h	n.c.	n.c.
HEC-1-A 10nM E1	n.c.	n.c.	n.c.	n.c.	7.083 nM	8.081 fmol/Mio/h	23.469 fmol/Mio/h	1.398 fmol/Mio/h	n.c.	n.c.
HEC-1-A 10nM E1	n.c.	n.c.	n.c.	n.c.	6.789 nM	8.859 fmol/Mio/h	31.603 fmol/Mio/h	1.538 fmol/Mio/h	n.c.	n.c.
HEC-1-A 100nM E1	n.c.	n.c.	n.c.	n.c.	70.124 nM	25.407 fmol/Mio/h	259.761 fmol/Mio/h	12.216 fmol/Mio/h	n.c.	n.c.
HEC-1-A 100nM E1	n.c.	n.c.	n.c.	n.c.	71.304 nM	34.643 fmol/Mio/h	304.473 fmol/Mio/h	15.599 fmol/Mio/h	n.c.	n.c.
HEC-1-A 500nM E1	n.c.	n.c.	n.c.	n.c.	355.688 nM	168.685 fmol/Mio/h	1348.447 fmol/Mio/h	77.748 fmol/Mio/h	n.c.	n.c.
HEC-1-A 500nM E1	n.c.	n.c.	n.c.	n.c.	321.471 nM	205.188 fmol/Mio/h	2076.345 fmol/Mio/h	109.444 fmol/Mio/h	n.c.	n.c.
HEC-1-A 1000nM E1	n.c.	n.c.	n.c.	n.c.	648.344 nM	313.068 fmol/Mio/h	3438.205 fmol/Mio/h	192.607 fmol/Mio/h	n.c.	n.c.
HEC-1-A 1000nM E1	n.c.	n.c.	n.c.	n.c.	645.325 nM	388.869 fmol/Mio/h	4096.289 fmol/Mio/h	227.757 fmol/Mio/h	n.c.	n.c.
HEC-1-A control	n.c.	n.c.	n.c.	n.c.	n.c.	n.c.	n.c.	n.c.	n.c.	n.c.
HEC-1-A control	n.c.	n.c.	n.c.	n.c.	n.c.	n.c.	n.c.	n.c.	n.c.	n.c.
HEC-1-A 10nM DHEA-S	n.c.	8.146 nM	n.c.	n.c.	n.c.	n.c.	n.c.	n.c.	n.c.	n.c.
HEC-1-A 10nM DHEA-S	n.c.	7.921 nM	n.c.	n.c.	n.c.	n.c.	n.c.	n.c.	n.c.	n.c.
HEC-1-A 100nM DHEA-S	n.c.	89.308 nM	n.c.	n.c.	n.c.	n.c.	n.c.	n.c.	n.c.	n.c.
HEC-1-A 100nM DHEA-S	n.c.	86.140 nM	n.c.	n.c.	n.c.	n.c.	n.c.	n.c.	n.c.	n.c.
HEC-1-A 500nM DHEA-S	n.c.	449.489 nM	n.c.	n.c.	n.c.	n.c.	n.c.	n.c.	n.c.	n.c.
HEC-1-A 500nM DHEA-S	n.c.	432.201 nM	n.c.	n.c.	n.c.	n.c.	n.c.	n.c.	n.c.	n.c.
HEC-1-A 1000nM DHEA-S	793.913 fmol/Mio/h	673.400 nM	n.c.	n.c.	n.c.	n.c.	n.c.	n.c.	n.c.	n.c.
HEC-1-A 1000nM DHEA-S	1302.654 fmol/Mio/h	607.171 nM	n.c.	n.c.	n.c.	n.c.	n.c.	n.c.	n.c.	n.c.
HEC-1-A 10nM E1-S	n.c.	n.c.	n.c.	n.c.	31.455 fmol/Mio/h	5.997 nM	n.c.	0.592 fmol/Mio/h	n.c.	n.c.
HEC-1-A 10nM E1-S	n.c.	n.c.	n.c.	n.c.	38.320 fmol/Mio/h	7.783 nM	n.c.	0.771 fmol/Mio/h	n.c.	n.c.
HEC-1-A 100nM E1-S	n.c.	n.c.	n.c.	n.c.	345.704 fmol/Mio/h	64.799 nM	53.507 fmol/Mio/h	4.569 fmol/Mio/h	n.c.	n.c.
HEC-1-A 100nM E1-S	n.c.	n.c.	n.c.	n.c.	300.360 fmol/Mio/h	65.131 nM	45.295 fmol/Mio/h	4.082 fmol/Mio/h	n.c.	n.c.
HEC-1-A 500nM E1-S	n.c.	n.c.	n.c.	n.c.	1757.155 fmol/Mio/h	323.986 nM	252.669 fmol/Mio/h	20.533 fmol/Mio/h	n.c.	n.c.
HEC-1-A 500nM E1-S	n.c.	n.c.	n.c.	n.c.	1855.583 fmol/Mio/h	419.430 nM	257.309 fmol/Mio/h	43.819 fmol/Mio/h	n.c.	n.c.
HEC-1-A 1000nM E1-S	n.c.	n.c.	n.c.	n.c.	12910.448 fmol/Mio/h	388.136 nM	2100.170 fmol/Mio/h	74.554 fmol/Mio/h	n.c.	n.c.

n.c. – not calculable due to levels below the limit of detection or below the limit of quantification.

Table S4. Results of immunohistochemistry presented as scores.

	IHC scores					
**Tissue sample**	**ABCG2 control**	**ABCG2 tumor**	**OSTβ control**	**OSTβ tumor**	**OATP1B3 control**	**OATP1B3 tumor**
2	NA	100	NA	60	NA	45
3	100	100	100	90	135	90
6	NA	90	NA	80	NA	135
7	NA	100	NA	20	NA	NA
9	100	100	150	75	180	225
10	100	100	100	95	135	180
11	NA	100	NA	130	NA	158
14	NA	100	NA	70	NA	180
15	NA	100	NA	8	NA	135
16	NA	100	NA	98	NA	135
18	NA	90	NA	8	NA	45
19	100	70	100	20	135	60
20	NA	60	NA	50	NA	43
21	NA	100	NA	85	NA	180
22	100	NA	100	NA	135	NA
23	NA	78	NA	48	NA	88
24	NA	90	NA	14	NA	53
25	100	50	100	60	180	135
26	100	100	100	23	135	90
30	NA	90	NA	0	NA	40
31	NA	85	NA	55	NA	113
33	NA	100	NA	88	NA	180
34	NA	100	NA	65	NA	40
35	100	100	100	85	135	50
38	100	98	100	30	180	180
39	NA	100	NA	98	NA	40
40	NA	100	NA	95	NA	135
44	100	NA	100	NA	180	NA
46	100	45	100	80	135	135
47	100	95	98	80	113	103
48	NA	100	NA	120	NA	135
49	100	35	100	90	135	90
50	NA	90	NA	80	NA	90
51	NA	70	NA	40	NA	160
52	NA	83	NA	82	NA	68
53	100	NA	100	NA	180	NA
54	100	55	100	35	158	33
55	NA	21	NA	7	NA	88
56	100	50	100	80	135	90
57	100	100	100	90	68	90
58	100	NA	100	NA	158	NA
60	100	NA	100	NA	135	NA
61	100	15	100	20	135	10
62	100	100	100	100	158	135
63	100	98	100	80	135	10
64	100	75	100	70	135	30
65	100	NA	100	NA	113	NA
66	NA	90	NA	80	NA	90
68	NA	100	NA	90	NA	90
69	NA	80	NA	80	NA	30
70	100	75	100	0	135	180
71	100	85	100	13	180	180

NA – data not available.
